# A novel mucosal bivalent vaccine of EV-A71/EV-D68 adjuvanted with polysaccharides from *Ganoderma lucidum* protects mice against EV-A71 and EV-D68 lethal challenge

**DOI:** 10.1186/s12929-023-00987-3

**Published:** 2023-12-18

**Authors:** Yu-Li Lin, Pei-Yun Cheng, Chiao-Li Chin, Kuan-Ting Chuang, Jing-Yi Lin, Ning Chang, Chun-Kei Pan, Cheng-Sheng Lin, Siao-Cian Pan, Bor-Luen Chiang

**Affiliations:** 1 Department of Medical Research, National Taiwan University Hospital, Taipei, Taiwan; 2 Graduate Institute of Immunology, College of Medicine, National Taiwan University, Taipei, Taiwan; 3 Department of Clinical Laboratory Sciences and Medical Biotechnology, College of Medicine, National Taiwan University, Taipei, Taiwan; 4 Department of Pediatrics, National Taiwan University Hospital, Taipei, Taiwan

**Keywords:** Acute flaccid myelitis, Acute flaccid paralysis, Adjuvant, Enterovirus A71, Enterovirus D68, Intranasal, Mucosal vaccine, *Ganoderma lucidum* polysaccharide

## Abstract

**Background:**

Human enteroviruses A71 (EV-A71) and D68 (EV-D68) are the suspected causative agents of hand-foot-and-mouth disease, aseptic meningitis, encephalitis, acute flaccid myelitis, and acute flaccid paralysis in children. Until now, no cure nor mucosal vaccine existed for EV-A71 and EV-D68. Novel mucosal bivalent vaccines are highly important for preventing EV-A71 and EV-D68 infections.

**Methods:**

In this study, formalin-inactivated EV-A71 and EV-D68 were used as antigens, while PS-G, a polysaccharide from *Ganoderma lucidum*, was used as an adjuvant. Natural polysaccharides have the characteristics of intrinsic immunomodulation, biocompatibility, low toxicity, and safety. Mice were immunized intranasally with PBS, EV-A71, EV-D68, or EV-A71 + EV-D68, with or without PS-G as an adjuvant.

**Results:**

The EV-A71 + EV-D68 bivalent vaccine generated considerable EV-A71- and EV-D68-specific IgG and IgA titres in the sera, nasal washes, saliva, bronchoalveolar lavage fluid, and feces. These antibodies neutralized EV-D68 and EV-A71 infectivity. They also cross-neutralized infections by different EV-D68 and EV-A71 sub-genotypes. Furthermore, compared with the PBS group, EV-A71 + EV-D68 + PS-G-vaccinated mice exhibited an increased number of EV-D68- and EV-A71-specific IgA- and IgG-producing cells. In addition, T-cell proliferative responses, and IFN-γ and IL-17 secretion in the spleen were substantially induced when PS-G was used as an adjuvant with EV-A71 + EV-D68. Finally, in vivo challenge experiments demonstrated that the immune sera induced by EV-A71 + EV-D68 + PS-G conferred protection in neonate mice against lethal EV-A71 and EV-D68 challenges as indicated by the increased survival rate and decreased clinical score and viral RNA tissue expression. Taken together, all EV-A71/EV-D68 + PS-G-immunized mice developed potent specific humoral, mucosal, and cellular immune responses to EV-D68 and EV-A71 and were protected against them.

**Conclusions:**

These findings demonstrated that PS-G can be used as a potential adjuvant for EV-A71 and EV-D68 bivalent mucosal vaccines. Our results provide useful information for the further preclinical and clinical development of a mucosal bivalent enterovirus vaccine against both EV-A71 and EV-D68 infections.

**Graphical Abstract:**

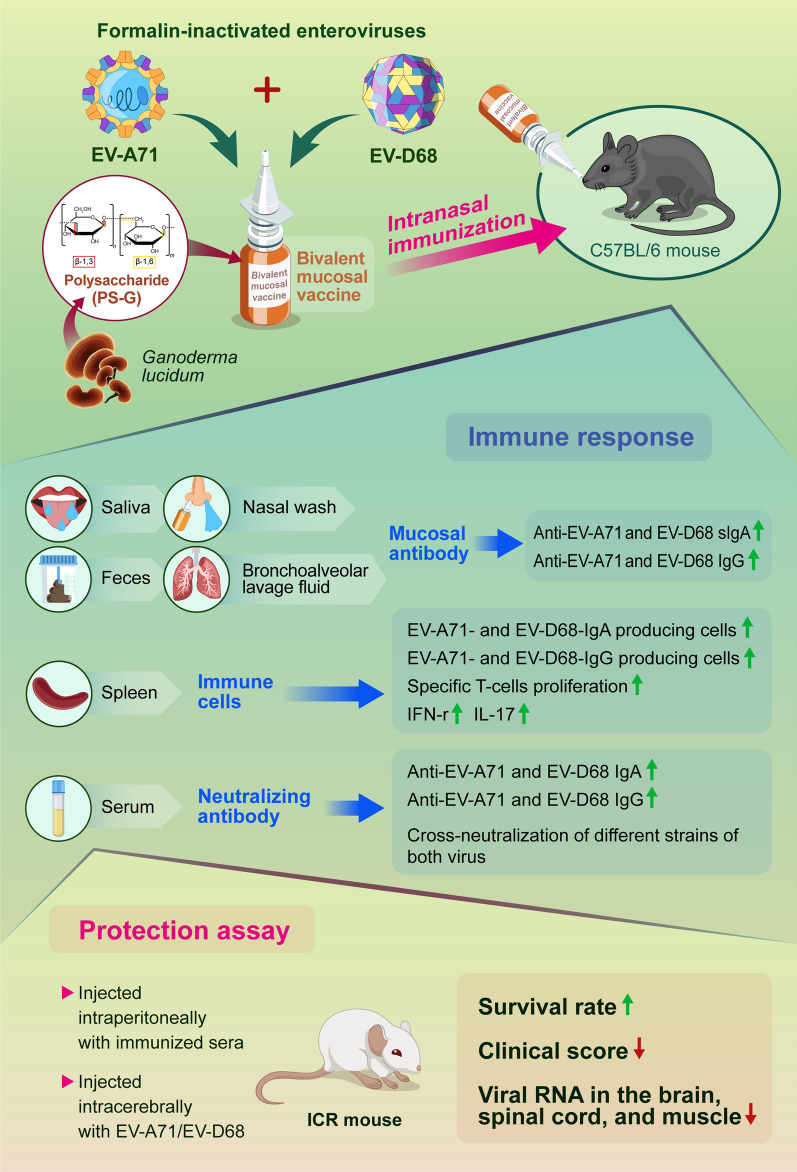

**Supplementary Information:**

The online version contains supplementary material available at 10.1186/s12929-023-00987-3.

## Background

Enterovirus A71 (EV-A71) and D68 (EV-D68) belong to the *Picornaviridae* family. They are non-enveloped, positive-sense, single-stranded RNA viruses composed of four structural proteins: VP1, VP2, and VP3 comprising the outer surface of the capsid, and VP4 located on the internal surface of the capsid shell [1–3]. EV-A71 and EV-D68 belong to the enterovirus A and D serotypes, respectively [4].

EV-A71 was first identified in an infant with encephalitis in California in 1969 [5]. Since then, EV-A71 has repeatedly caused large-scale outbreaks in the Asia–Pacific region, including Brunei, Cambodia, China, Malaysia, Singapore, Taiwan, and Vietnam, with some studies suggesting the occurrence of cyclical epidemics every 2–3 years [6]. Human EV-A71 infection can cause hand-foot-and-mouth disease (HFMD), herpangina, brainstem encephalitis, acute flaccid paralysis (AFP), neurological pulmonary oedema, cardiopulmonary dysfunction, and even death. In 1998, a large EV-A71 outbreak in Taiwan led to an epidemic involving 129,106 cases of HFMD or herpangina, resulting in 405 severe cases and 78 deaths (91% of whom were children aged ≤ 5 years old) [7]. EV-A71 can be transmitted via the faecal-oral route or through direct or indirect contact with respiratory droplets or pollutants. Of note, viral shedding can persist for approximately 2 weeks in the respiratory tract and up to three months after infection in the stool [8].

EV-D68 was initially isolated from children with pneumonia and bronchiolitis in California in 1962 [9]. A large outbreak of EV-D68-associated acute flaccid paralysis occurred in the USA and Canada in 2014 [10]. Currently, EV-D68 circulates regularly in the United States and is associated with severe respiratory diseases and acute flaccid myelitis, which is similar to poliomyelitis [11]. Acute flaccid myelitis (AFM) affects the grey matter of the spinal cord, weakening the muscles and reflexes of the body. EV-D68 has been recognized as a re-emerging pathogen [12], with cyclical epidemics occurring every 2 years. EV-D68 shares many physiochemical properties with human rhinoviruses, including enhanced replication at 33 °C, which is the temperature in the nasal cavity [13]. EV-D68 has been mainly isolated from respiratory samples, whereas faecal isolation has rarely been reported [14, 15]. Therefore, transmission of EV-D68 is thought to occur primarily via the respiratory route rather than the faecal-oral route, which is the predominant mode of transmission for most other enteroviruses.

In the past 3 years, outbreaks of infectious diseases transmitted through droplets and contact have been reduced owing to the active adherence of people to COVID-19 pandemic prevention policies and the implementation of pandemic prevention regulations, such as frequent hand washing, wearing masks, and maintaining social distancing. However, as the COVID-19 pandemic continues to slow down and more enterovirus-susceptible hosts accumulate, the epidemic risk of EV-A71 and EV-D68 rises and cannot be ignored as the management of EV-A71 and EV-D68 outbreaks relies solely on public monitoring. Currently, no specific drugs are available against EV-A71 or EV-D68, and while EV-A71 vaccines have been developed in China and Taiwan [16, 17], an EV-D68 vaccine is yet to be developed. An effective vaccine against EV-A71 and EV-D68 could prevent virus-induced morbidity and mortality.

Mucosal vaccines are delivered through the mucosal surfaces of the body, including the nose, mouth, and genital tract. Mucosal vaccines stimulate the immune system at these surfaces, which are common sites of entry for many pathogens, including enteroviruses. Mucosal vaccines can efficiently induce secretory IgA from mucosal surfaces, thereby preventing and limiting infections at the site of viral entry. An ideal mucosal adjuvant would increase the residence time of antigens and secretion of mucosal antibodies while reducing the dose of antigens required. Accordingly, nasal inoculation with appropriate adjuvants has attracted increasing interest owing to its potential to establish robust mucosal immune responses while causing little pain or fear, especially in children and neonates [18].

*Ganoderma lucidum*, also known as Reishi mushroom, has been widely used as a natural medicine in China and other Asian countries owing to its immune regulatory function [19, 20]. The polysaccharide from *G. lucidum* (PS-G) contains a branched (1→6)-β-d-glucan moiety. We have previously confirmed that PS-G can effectively increase the activation and maturation of immature dendritic cells (DCs) [21], when compared with aluminium salts (alum), resulting in a Th1 response characterized by the secretion of IFN-γ and generation of anti-ovalbumin IgG2a when used PS-G as an adjuvant in BALB/c mice [22]. In 2020, we demonstrated that PS-G-adjuvanted EV-A71 promoted a potent immune response against EV-A71 infections [23]. Recently, alum-adjuvanted inactivated EV-A71 vaccines have been approved in China and Taiwan [24, 25]. However, these vaccine are administered intramuscularly. We hope to develop effective mucosal vaccines against EV-A71 and EV-D68 for children. In this study, we used PS-G as an adjuvant in EV-A71 and EV-D68 bivalent mucosal vaccines administered via the nasal route and studied the immune responses of the host.

Our study suggested that the mucosal bivalent inactivated EV-A71/EV-D68 vaccine can induce the generation of neutralizing antibodies against EV-A71 and EV-D68, making it effective against EV-A71 and EV-D68 infections in mice. Overall, our results showed that the use of PS-G as an adjuvant in EV-A71/EV-D68 nasal vaccines promoted a strong mucosal response and systemic immunity, suggesting that PS-G may be useful as an effective adjuvant for the development of intranasal EV-A71/EV-D68 bivalent mucosal vaccines.

## Methods

### Viruses and vaccines

In this study, the EV-A71 strain TW/2272/98 (C2 genogroup) for the purification of the inactivated EV-A71 vaccine was kindly provided by Prof. Shin-Ru Shih, at the Research Center for Emerging Viral Infections, Chang Gung University, Taiwan. EV-D68 (CDC_NO 2010-01788), in Clade B3, was used as the vaccine antigen. The EV-A71 strains 200307025 (B4 genogroup, isolated in 2003) and 200802571 (C4 genogroup, isolated in 2008), and EV-D68 strains (CDC_NO 2010-01788 and CDC_NO 2016-05298) were obtained from the Centers for Disease Control, Taiwan. The EV-D68 US/MO/14-18947 (MO/47) strain was provided by Dr. Jim-Tong Horng of the Department of Biochemistry and Molecular Biology, Chang Gung University, Taiwan. The EV-A71 TW/4643/MP4 strain (MP4, genotype C2) with increased virulence in mice was kindly provided by Dr. Jen-Ren Wang of the National Cheng Kung University, Tainan, Taiwan [26]. Both EV-A71 and EV-D68 were propagated in human rhabdomyosarcoma (RD) cells (ATCC No. CCL-136) cultured in Minimum Essential Medium Alpha (α-MEM; HyClone), at 37 °C (EV-A71) or 33 °C (EV-D68). EV-A71 and EV-D68 were purified as previously described [27].

### Purification of PS-G from G. lucidum

Using a previously described method [28], the fruiting bodies of *G. lucidum* were washed, disintegrated, and extracted using boiling double-distilled water. The obtained crude polysaccharide was passed through a gel-filtration Sephadex G 50 column and further purified through anion-exchange chromatography using a diethylaminoethyl cellulose column [19]. PS-G is a protein-bound polysaccharide consisting of 95% polysaccharides and 5% proteins. Monosaccharide composition analysis by nuclear magnetic resonance (NMR) using a polysaccharide component assay kit from SugarLighter (Taiwan) showed that PS-G was mainly composed of glucose (79%) and mannose (21%). Diffusion-ordered nuclear magnetic resonance spectroscopy (DOSY NMR) using the SugarLighter kit determined that the molecular weight of PS-G was approximately 15 kDa [23].

### Immunization of mice

Female C57BL/6 mice (6-week-old) were intranasally (i.n.) immunized with 12 μL aliquots (6 μL/nostril) of the vaccine containing 2.5 μg formalin-inactivated EV-A71, 2.5 μg formalin-inactivated EV-D68, or 2.5 μg EV-A71 and 2.5 μg EV-E68 with or without 10 μg PS-G or 20 μg CpG (ODN2395, type C; Invivogen) as an adjuvant; the mixture was slowly dropped into each nostril to prevent asphyxia. All mice were vaccinated thrice at 3-week intervals. Serum samples were collected 2 weeks after the third vaccination and stored at − 80 °C for monitoring the immune response. Nasal washes, saliva, bronchoalveolar lavage fluid (BALF), and fecal samples were collected to measure mucosal IgA and IgG titres. All animal studies were approved by the Institutional Animal Care and Use Committee (Approval No: 20160347) at the College of Medicine, National Taiwan University (Taipei, Taiwan) and performed in accordance with the approved guidelines.

### Detection of anti-virus-specific IgA, IgG, IgG1, and IgG2c

As shown in our previous study [29], microplates (Nunc) were coated overnight with 5 μg/mL inactivated EV-D68 or EV-A71, followed by blocking in Tris-buffered saline with Tween-20 (TBST) supplemented with 1% bovine serum albumin (BSA). Serum, nasal washes, saliva, BALF, or faecal extracts were then added to microplates for 2 h at 25 °C. The plates were washed and incubated with goat anti-mouse IgA-HRP (1:5000, Bethyl), anti-mouse IgG-HRP, IgG1-HRP, or IgG2c-HRP (1:10,000, Bethyl) antibodies. Subsequently, 3,3′,5,5′-tetramethylbenzidine substrate was added for colour development and the reaction was stopped by adding 100 μL of 1 M H_2_SO_4_. Absorbance was measured at 450 and 550 nm using a SpectraMax iD5 Multi-Mode Microplate Reader (Molecular Devices, San Jose, CA, USA).

### Neutralization titre assay

Serum samples were heat-inactivated at 56 °C for 30 min and then two-fold serially diluted sera were mixed with an equal volume of 100-fold TCID_50_ EV-D68 or EV-A71 in a 96-well plate and incubated for 1 h at 37 °C. Next, 2 × 10^4^ RD cells grown in α-MEM supplemented with 2% foetal bovine serum (FBS) were added to the mixture, and the cytopathic effect was determined after 4 d. The highest serum dilution that resulted in no cytopathic effects was identified as the neutralization titre.

### Enzyme-linked ImmunoSpot (ELISPOT) assay

Microplates (Millipore, Bedford, MA, USA) were coated overnight with purified EV-D68 or EV-A71 virions (10 μg/mL) and blocked with BSA. Spleen cells (5 × 10^5^) grown in RPMI-1640 supplemented with 10% FBS were added and incubated for 24 h at 37 °C and 5% CO_2_. The plates were then washed and incubated with HRP-conjugated goat anti-mouse IgG (1:000, Bethyl) or IgA (1:000, Bethyl). After 2 h of incubation, the HRP conjugate was removed and cells were washed. Next, 100 μL 3-amino-9-ethylcarbazole substrate (Becton, Dickinson and Company, Franklin Lakes, NJ, USA) was added, and the spots were allowed to develop for 10 min. After completion of the ELISPOT assay, the plates were scanned and analysed using an ImmunoSpot S6 UV Reader (Cellular Technology Limited, Cleveland, OH, USA). The spots were counted using ImmunoSpot v.6.0. Spots from four wells with two million spleen cells were calculated as one set of data.

### T-cell response assay

Single cell suspensions from the spleens of immunized mice were stimulated with inactivated EV-D68 or EV-A71 (10 μg/mL) in RPMI-1640 supplemented with 10% FBS, concanavalin A (2 μg/mL) as the positive control, or medium only as the negative control. For cytokine analyses, cells were cultured at 37 °C for 3 d, and then the supernatants were harvested to detect the levels of interferon-γ (IFN-γ), interleukin-4 (IL-4), and IL-17 (R&D Systems, Minneapolis, MN, USA). For the proliferation assay, cells were cultured for 5 d and pulsed with [^3^H]-thymidine (Amersham Biosciences) for 18 h at 37 °C and 5% CO_2_. After harvesting the cells, thymidine incorporation was measured using a scintillation counter (TopCount NXT Scintillation and Luminescence Counter; PerkinElmer).

### Flow cytometry analysis

Splenocytes were isolated from all mice and cultured with EV-D68 or EV-A71 for 5 d. After incubation, cells were treated with phorbol 12-myristate 13-acetate, ionomycin, and monensin for 4 h. Cells were collected, washed, treated with Fc blocker for 15 min, and stained with anti-CD4 and anti-CD8 antibodies for 30 min at 4 °C. Cells were washed, fixed, permeabilized using a permeabilization buffer, and stained with anti-IFN-γ and anti-IL-17 antibodies for 1 h. Finally, the number of cells was determined using a FACSCalibur flow cytometer (BD Biosciences, San Jose, CA, USA).

### In vivo protection assay

The protective efficacy of the inactivated EV-D68 and EV-A71 bivalent vaccines was tested using a passive serum transfer assay. Briefly, 2-d-old ICR mice were intraperitoneally (i.p.) injected with 20 μL of immunized sera. After 6 h, the suckling mice were intracerebrally (i.c.) injected with RD medium, 7.4 × 10^6^ PFU of EV-D68 (5298), or 5 × 10^6^ PFU of EV-A71 (MP4, the fourth passage of the mouse-adapted EV-A71 strain from EV-A71 TW/4643/98). The survival and clinical scores of mice were monitored daily for 14 d. Clinical scores were defined as follows: 0, healthy; 1, wasting; 2, limb weakness; 3, paralysis in only one limb; 4, paralysis in 2–4 limbs; and 5, death.

### Determination of viral loads in tissues

Organs from ICR mice were harvested and homogenized in phosphate-buffered saline (PBS) containing protease inhibitors. Total RNA was extracted using the TRIzol reagent (Life Technologies, Carlsbad, CA, USA) and chloroform. RNA was precipitated using isopropanol and 75% ethanol. For reverse transcription, cDNA was synthesised using a high-capacity cDNA RT kit (Life Technologies) in a Biometra T 3000 Thermocycler. For amplification of the DNA signal, real-time PCR was performed using the SYBR green Supermix (Bio-Rad, Hercules, CA, USA) in a Bio-Rad CFX Connect system (Bio-Rad). The sequences of the cDNA primer pairs used were as follows: GAPDH (Forward: 5′-GTTCCTACCCCCAATGTG-3′; Reverse: 5′-CAACCTGGTCCTCAGTGTAG-3′), EV-A71 VP1 (Forward: 5′-CTGGTAAAGGTCCAGCACTC-3′; Reverse: 5′-GGGAGGTCTATCTCTCCAAC-3′), and EV-D68 VP1 (Forward: 5′-CAAACTCGCACAGTGATAAAYCARCA-3′; Reverse: 5′-GTATTATTACTACTACCATTCACNGCNAC-3′). The levels of expression of the VP1 gene were normalized to those of *GAPDH*.

### Statistical analysis

The GraphPad Prism 9 Software was used for all data analyses. Student’s *t*-test and one-way ANOVA were used to compare results between different groups. *p* values < 0.05 were considered to be statistically significant.

## Results

### Long-term memory immunity induced by the EV-A71 or EV-D68 mucosal vaccine

**Fig. 1:**
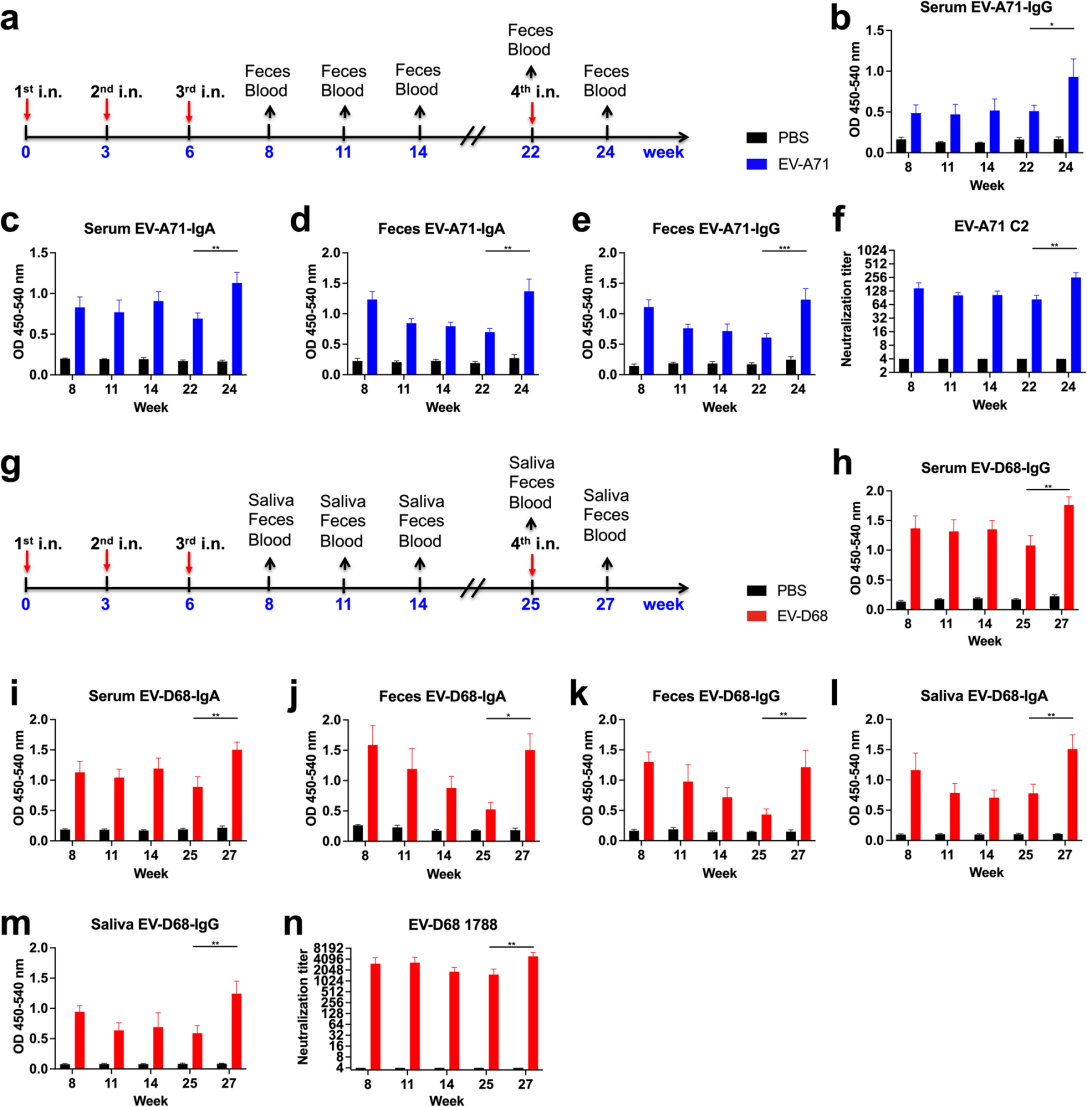
Long-term memory immunity induced by inactivated EV-A71 or EV-D68 mucosal vaccines in mice. Mice were intranasally immunized with PBS, formalin-inactivated EV-A71 (2.5 μg/mouse), or EV-D68 (2.5 μg/mouse) thrice at 3-week intervals. **a** Immunization schedules for the EV-A71 mucosal vaccine. Serum and faecal samples were collected from immunized mice at weeks 8, 11, 14, 22, and 24. The levels of EV-A71-specific IgG and IgA in the serum (**b**, **c**) and feces (**d**, **e**) were measured using ELISA. **f** Neutralization titre against EV-A71 C2 infection in the serum of immunized mice. **g** Immunization schedules for the EV-D68 mucosal vaccine. Serum, feces, and saliva samples were collected from immunized mice at weeks 8, 11, 14, 25, and 27. The levels of EV-D68-specific IgG and IgA in the serum (**h**, **i**), feces (**j**, **k**), and saliva (**l**, **m**) were measured using ELISA. **n** Neutralization titre against EV-D68 1788 infection in the serum of immunized mice. *p < 0.05; **p < 0.01; ***p < 0.001

To assess the enduring protective effect conferred by the EV-A71 or EV-D68 mucosal vaccines, mice were i.n. immunized with PBS, 2.5 μg formalin-inactivated EV-A71, or 2.5 μg formalin-inactivated EV-D68 according to the schedule illustrated in Fig. 1a, g. Compared with the PBS group, we observed considerable elevations in the levels of EV-A71-specific IgG and IgA in both serum and faecal samples, even at week 22 (Fig. 1b–e). At week 22, we administered the fourth intranasal immunization with EV-A71, resulting in a significant increase in the levels of EV-A71-specific IgG and IgA in the serum and feces at week 24 compared with those at week 22 in the EV-A71 immunized group (p < 0.01). All mice immunized with inactivated EV-A71 vaccines developed antibodies capable of neutralizing EV-A71 C2 in the sera at all indicated time points (Fig. 1f). Moreover, mice vaccinated with EV-D68 exhibited heightened levels of EV-D68-specific IgG and IgA in the serum, feces, and saliva, even at week 25 (Fig. 1h–m). At week 25, we administered the fourth intranasal immunization with EV-D68, leading to a considerable elevation in the levels of EV-D68-specific IgG and IgA in the serum, feces, and saliva at week 27 compared with those at week 25 in the EV-D68 immunized group (p < 0.01). Notably, all mice immunized with inactivated EV-D68 vaccinations generated antibodies capable of neutralizing EV-D68 1788 in the sera at all indicated time points (Fig. 1n). These results suggested that intranasal administration of EV-A71 or EV-D68 generates and maintains EV-A71 or EV-D68-specific antibodies, both locally in feces or saliva and systemically in the serum, for a minimum of 16 or 19 weeks following the third immunization, signifying the potential long-term memory immunity induced by EV-A71 or EV-D68 mucosal vaccines.

### *Mucosal EV-D68- and EV-A71-specific IgA and IgG responses following immunization with the EV-A71* + *EV-D68 bivalent vaccine*

**Fig. 2:**
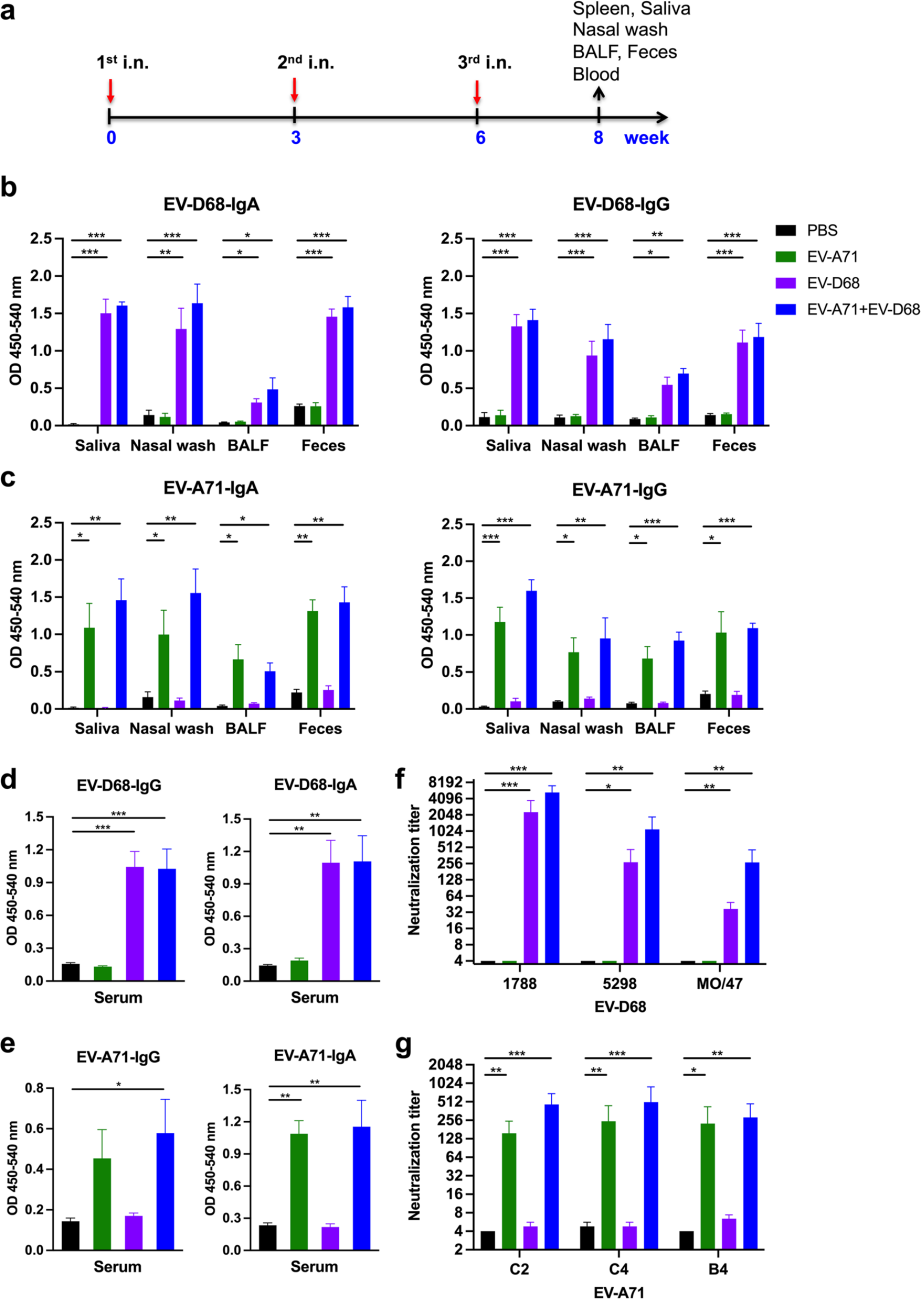
Effect of the EV-A71 + EV-D68 bivalent vaccine on EV-D68- and EV-A71-specific IgG and IgA and neutralization titres against infection by different enteroviruses in immunized mice. Mice were intranasally immunized with PBS, formalin-inactivated EV-A71 (2.5 μg/mouse), formalin-inactivated EV-D68 (2.5 μg/mouse), and combined formalin-inactivated EV-A71 (2.5 μg/mouse) and EV-D68 (2.5 μg/mouse) thrice at 3-week intervals. **a** Three-dose immunization schedules. **b** The levels of EV-D68-specific IgA and IgG and **c** EV-A71-specific IgA and IgG in the saliva, nasal wash, BALF, and feces of mice after the third immunization were measured using ELISA. **d** The levels of EV-D68-specific IgG and IgA and **e** EV-A71-specific IgG and IgA in the sera of mice after the third intranasal immunization were measured using ELISA. Sera were serially diluted (2^3^–2^12^), mixed with **f** EV-D68 and **g** EV-A71 virus, and used to infect RD cells. After 4 d, the the highest dilution that resulted in the virus producing no cytopathic effect was considered to be the neutralization titre. All data are expressed as the mean ± SEM. *p < 0.05, **p < 0.01, ***p < 0.001

Mice were i.n. immunized thrice at 3-week intervals (Fig. 2a) with PBS, 2.5 μg EV-A71, 2.5 μg EV-D68, or 2.5 μg EV-A71 plus 2.5 μg EV-E68. We observed that compared with the PBS group, mice immunized with inactivated EV-D68 alone or EV-A71 + EV-D68 generated a considerable amount of EV-D68-specific IgA and IgG in the saliva, nasal wash, BALF, and feces after the third vaccination (Fig. 2b). Likewise, compared with the PBS group, mice immunized with inactivated EV-A71 alone or EV-A71 + EV-D68 produced a considerable amount of EV-A71-specific IgA and IgG in the nasal wash, saliva, BALF, and feces after the third vaccination (Fig. 2c).

### *EV-A71* + *EV-D68 bivalent vaccine elicited specific IgG, IgA, and neutralizing antibodies in mice sera*

We found that compared with the PBS group, mice vaccinated with EV-D68 alone or the EV-A71 + EV-D68 bivalent vaccine generated a considerable amount of EV-D68-specific IgG and IgA in the sera after the third intranasal immunization (Fig. 2d). Similarly, compared with the PBS group, mice vaccinated with EV-A71 alone or the EV-A71 + EV-D68 bivalent vaccine generated a considerable amount of EV-A71-specific IgG and IgA in the sera after the third intranasal immunization (Fig. 2e).

To determine whether the inactivated EV-D68 and EV-A71 vaccines could elicit functional anti-EV-D68 and anti-EV-A71 antibodies, we analysed the neutralizing antibody titre in the sera after the third vaccination using a TCID_50_-reduction assay in RD cells. Briefly, we serially diluted sera two-fold and cultured it with EV-D68 or EV-A71 and RD cells, and then determined the neutralizing titre as the highest dilution that neutralized 100-fold TCID_50_ of EV-D68 or EV-A71 virus, leading to no cytopathic effect in enterovirus-sensitive RD cells. We observed that all mice immunized with inactivated EV-D68 or EV-A71 + EV-D68 generated antibodies capable of neutralizing different EV-D68 sub-genotypes in the sera after the third vaccination (Fig. 2f). Likewise, mice vaccinated with inactivated EV-A71 or EV-A71 + EV-D68 generated antibodies capable of neutralizing different EV-A71 sub-genotypes in the sera after the third vaccination (Fig. 2g).

### Cellular immune responses in EV-A71 and EV-D68-vaccinated mice

**Fig. 3:**
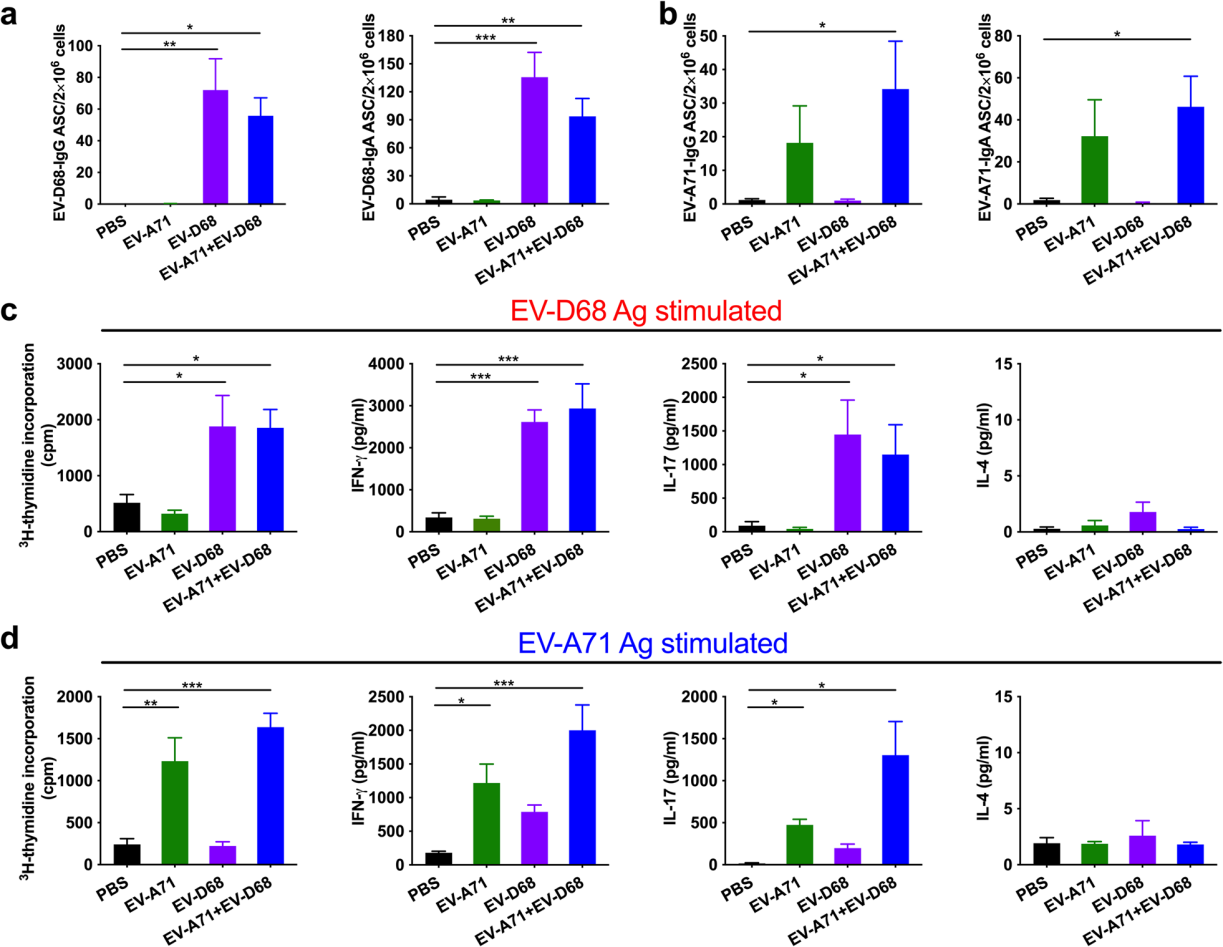
Effect of the EV-A71 + EV-D68 bivalent vaccine on the number of antibody-secreting cells (ASCs), T-cell proliferation, and cytokine release in splenocytes. Mice were intranasally immunized with PBS, formalin-inactivated EV-A71 (2.5 μg/mouse) alone, formalin-inactivated EV-D68 (2.5 μg/mouse) alone, and combined formalin-inactivated EV-A71 (2.5 μg/mouse) with EV-D68 (2.5 μg/mouse) thrice at 3-week intervals. Spleen was isolated 2 weeks after the third immunization, and then the numbers of **a** EV-D68-specific IgG and IgA ASCs and **b** EV-A71-specific IgG and IgA ASCs were measured using ELISPOT. **c**, **d** Splenocytes from immunized mice were harvested and cultured in medium containing 10 μg/mL heat-inactivated EV-D68 or EV-A71. After culture for 5 d, proliferation was measured as [^3^H]-thymidine incorporation in splenocytes. Culture supernatants of splenocytes were analysed for levels of IFN-γ, IL-17, and IL-4 after 2 d in response to heat-inactivated EV-D68 or EV-A71. Thymidine uptake was determined by harvesting cells and using a scintillation counter to measure the level of incorporation (as counts per minute; cpm). All data are expressed as the mean ± SEM. *p < 0.05, **p < 0.01, ***p < 0.001

To further characterize the quality of memory responses induced by the EV-A71 + EV-D68 bivalent vaccines, we analysed the number of antibody-secreting B-cells in the spleen after the third vaccination. We found that compared with the PBS group, the amount of EV-D68-specific IgG and IgA antibody-secreting cells (ASCs) was substantially increased in mice vaccinated with EV-D68 alone or the EV-A71 + EV-D68 bivalent vaccine (Fig. 3a). The amount of EV-A71-specific IgG and IgA ASCs was considerably increased in mice immunized with EV-A71 alone or the EV-A71 + EV-D68 bivalent vaccine compared with the PBS group (Fig. 3b).

The strength of T helper cell responses plays an important role in the production of both humoral and cellular responses. We noticed the significantly enhanced proliferation and activation of T-cells in splenocytes stimulated with the EV-D68 antigen in mice immunized with EV-D68 alone (*p* < 0.05) or with the EV-A71 + EV-D68 bivalent vaccine (*p* < 0.05) (Fig. 3c). We also measured cytokine production in splenocytes as an indicator of memory T-cell response. In particular, we detected that cells stimulated with the EV-D68 antigen from mice vaccinated with EV-D68 alone or the EV-A71 + EV-D68 bivalent vaccine produced significantly higher levels of IFN-γ (*p* < 0.001) and IL-17 (*p* < 0.05) than those in the PBS group (Fig. 3c). Similarly, splenocytes stimulated with the EV-A71 antigen from mice immunized with EV-A71 alone or the EV-A71 + EV-D68 bivalent vaccine exhibited enhanced proliferation and produced significantly higher levels of IFN-γ (*p* < 0.05, *p* < 0.001) and IL-17 (*p* < 0.05) than those in the PBS group (Fig. 3d). However, we observed very low levels of IL-4 that did not differ between groups. Overall, these data suggested that EV-D68 and EV-A71 can induce memory T-cell responses.

### In vivo protection assay of EV-A71 + EV-D68 bivalent vaccine

**Fig. 4:**
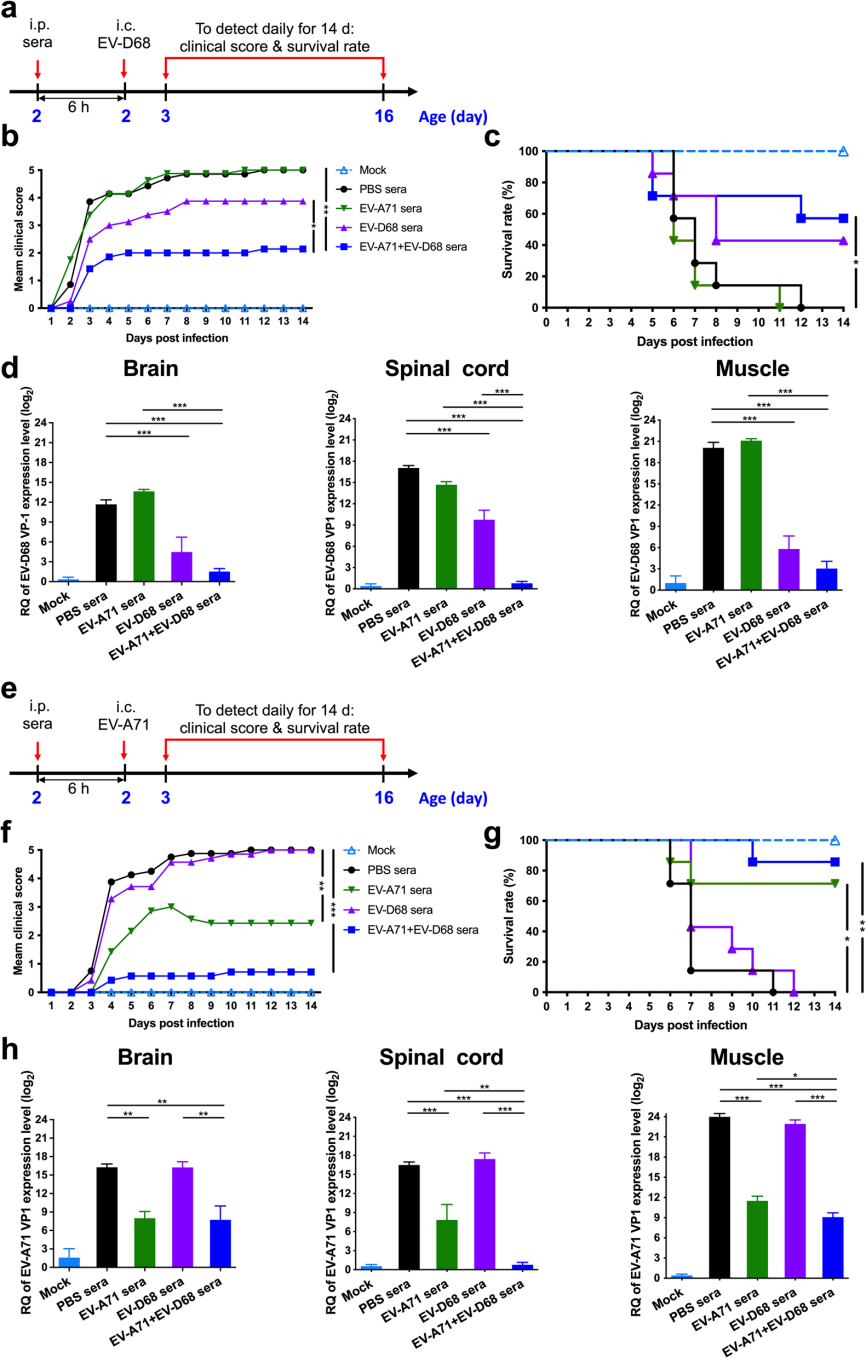
Protective efficacy of the inactivated EV-A71 + EV-D68 vaccine in the EV-D68 or EV-A71 infection mouse model. The passive sera transfer and virus challenge schedules. Briefly, 2-d-old ICR mice were i.p. injected with 20 μL anti-PBS, anti-EV-A71, anti-EV-D68, or anti-EV-A71 + EV-D68 sera. After 6 h, the suckling mice were intracerebrally (i.c.) administered with RD medium, **a** EV-D68 (CDC_NO 2016-05298), or **e** EV-A71 (MP4). Mock mice were administered medium only. Mice were monitored daily for **b**, **f** clinical score and **c**, **g** survival rate for 14 d following infection. Clinical scores were graded as described in “Methods”. **d**, **h** Brain, spinal cord, and muscle were collected after infection, and viral loads within the indicated tissues were determined using real-time quantitative PCR (qRT-PCR). The levels of expression were normalized to those of *GAPDH*. Data are presented as the mean ± SEM. *p < 0.05, **p < 0.01, ***p < 0.001

We evaluated the in vivo protective efficacy of the inactivated EV-A71 + EV-D68 bivalent vaccine using a passive serum transfer assay. Briefly, we i.p. injected 2-d-old ICR mice with anti-PBS, anti-EV-A71, anti-EV-D68, or anti-EV-A71 + EV-D68 serum. After 6 h, we i.c. injected the suckling mice with a lethal dose of EV-D68 (5298) (Fig. 4a). We observed the mice daily for 14 d to determine clinical scores and survival. We determined that the clinical score in the EV-A71 + EV-D68 bivalent vaccine group was significantly lower than that in the PBS (*p* < 0.01) and EV-D68 groups (*p* < 0.05) (Fig. 4b). As shown in Fig. 4c, mice receiving anti-PBS sera started to show disease symptoms at 2 d post-infection (dpi), with all of them dying within 12 dpi. In contrast, we found that the survival rates of immunized-sera-treated mice were 42.9% and 57.1% in the EV-D68 and EV-A71 + EV-D68 groups, respectively. To study the replication and distribution of EV-D68 in infected mice, we determined the viral loads in the brain, spinal cord, and muscles of EV-D68 (5298)-infected mice using real-time PCR. We detected that the levels of EV-D68 VP1-specific RNA in the brain, spinal cord, and muscles were significantly decreased in the EV-D68 and EV-A71 + EV-D68 groups (*p* < 0.001). Of note, the levels of EV-D68 VP1 specific RNA in these tissues in the EV-A71 + EV-D68 group was lower than that in the EV-D68 group. These results indicated that passive transfer of anti-EV-D68 or anti-EV-A71 + EV-D68 sera could provide effective protection against the lethal EV-D68 (5298) virus challenge in a mouse model.

We i.p. injected newborn ICR mice with anti-PBS, anti-EV-A71, anti-EV-D68, or anti-EV-A71 + EV-D68 sera, and then challenged them with a lethal dose of EV-A71 (MP4). As shown in Fig. 4e, mice i.c. injected with RD medium alone (mock) did not show any indicators of disease, and all survived. We detected that the clinical score in the EV-A71 or EV-A71 + EV-D68 bivalent vaccine groups was considerably lower than that in the PBS group during the 2-week observation period (Fig. 4f). Mice receiving anti-PBS sera started to show disease symptoms at 3 dpi, and all of them eventually died within 11 dpi. In contrast, the survival rates of all immunized-sera-treated mice were 71.4% (*p* < 0.05) and 85.7% (*p* < 0.001) in the EV-A71 and EV-A71 + EV-D68 group, respectively (Fig. 4g). To study the replication and distribution of EV-A71 in infected mice, we determined the viral loads in tissues from EV-A71 (MP4)-infected mice using real-time PCR. We found that the levels of EV-A71 VP1-specific RNA in the brain, spinal cord, and muscles were considerably decreased in the EV-A71 and EV-A71 + EV-D68 groups (Fig. 4h). These results indicated that passive transfer of anti-EV-A71 or anti-EV-A71 + EV-D68 sera could provide effective protection against lethal EV-A71 (MP4) virus challenge.

### *Mucosal EV-D68- and EV-A71-specific IgA and IgG responses to intranasal EV-A71* + *EV-D68 bivalent vaccine immunization using PS-G as an adjuvant*

**Fig. 5:**
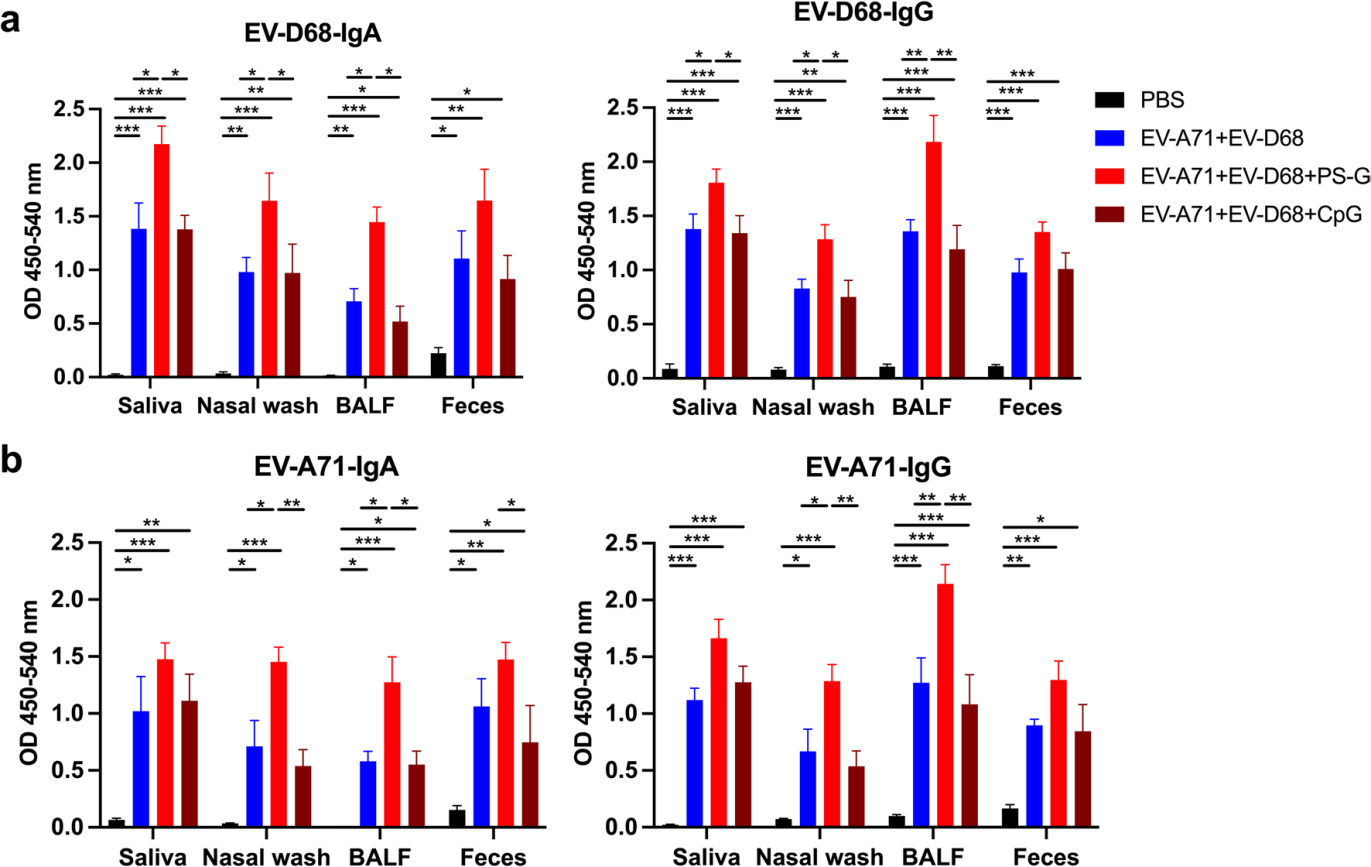
Effect of the EV-A71 + EV-D68 bivalent vaccine with or without PS-G or CpG as adjuvant on the levels of EV-D68-specific and EV-A71-specific IgA and IgG in the saliva, nasal wash, BALF, and feces of mice. Mice were intranasally immunized with PBS, formalin-inactivated EV-A71 (2.5 μg/mouse) and EV-D68 (2.5 μg/mouse), and formalin-inactivated EV-A71 (2.5 μg/mouse) and EV-D68 (2.5 μg/mouse) combined with PS-G (10 μg/mouse) or CpG (20 μg/mouse) as adjuvant thrice at 3-week intervals. The levels of **a** EV-D68-specific IgA and IgG, and **b** EV-A71-specific IgA and IgG in the saliva, nasal wash, BALF, and feces of mice after the third immunization were measured using ELISA. All data are expressed as the mean ± SEM. *p < 0.05, **p < 0.01, ***p < 0.001

We i.n. immunized mice thrice at 3-week intervals with PBS, EV-A71 (2.5 µg) plus EV-D68 (2.5 µg), with or without PS-G or CpG as an adjuvant. We observed that compared with the PBS group, EV-A71 + EV-D68, EV-A71 + EV-D68 + PS-G, and EV-A71 + EV-D68 + CpG vaccine-immunized mice generated a considerable amount of EV-D68-specific IgA and IgG (Fig. 5a) and EV-A71-specific IgA and IgG (Fig. 5b) in the saliva, nasal wash, BALF, and feces after the third vaccination. Likewise, compared with the PBS group, inactivated EV-A71 + EV-D68 and EV-A71 + EV-D68 + PS-G vaccine-immunized mice generated a considerable amount of EV-A71-specific IgA and IgG in mucosal tissues after the third vaccination (Fig. 5b). We further determined that the inactivated EV-A71 + EV-D68 vaccine using PS-G as an adjuvant induced higher levels of EV-D68-specific IgA and IgG in the saliva (*p* < 0.05), nasal wash (*p* < 0.05), and BALF (*p* < 0.05) (Fig. 5a), as well as higher levels of EV-A71-specific IgA and IgG in the nasal wash (*p* < 0.05) and BALF (*p* < 0.05) compared with those of the EV-A71 + EV-D68 vaccine (Fig. 5b) after the third vaccination. Moreover, we noticed that the EV-A71- and EV-D68-specific IgG and IgA titres in the saliva, nasal wash, BALF, and feces were substantially higher in the group injected with the bivalent vaccine with PS-G as an adjuvant than that with CpG. The utilization of CpG as an adjuvant yielded results akin to those observed in the group inoculated with the bivalent vaccine without an adjuvant.

### *EV-A71* + *EV-D68 bivalent vaccine using PS-G as an adjuvant elicited strong specific IgG, IgA, and neutralizing antibody responses in mice sera*

**Fig. 6:**
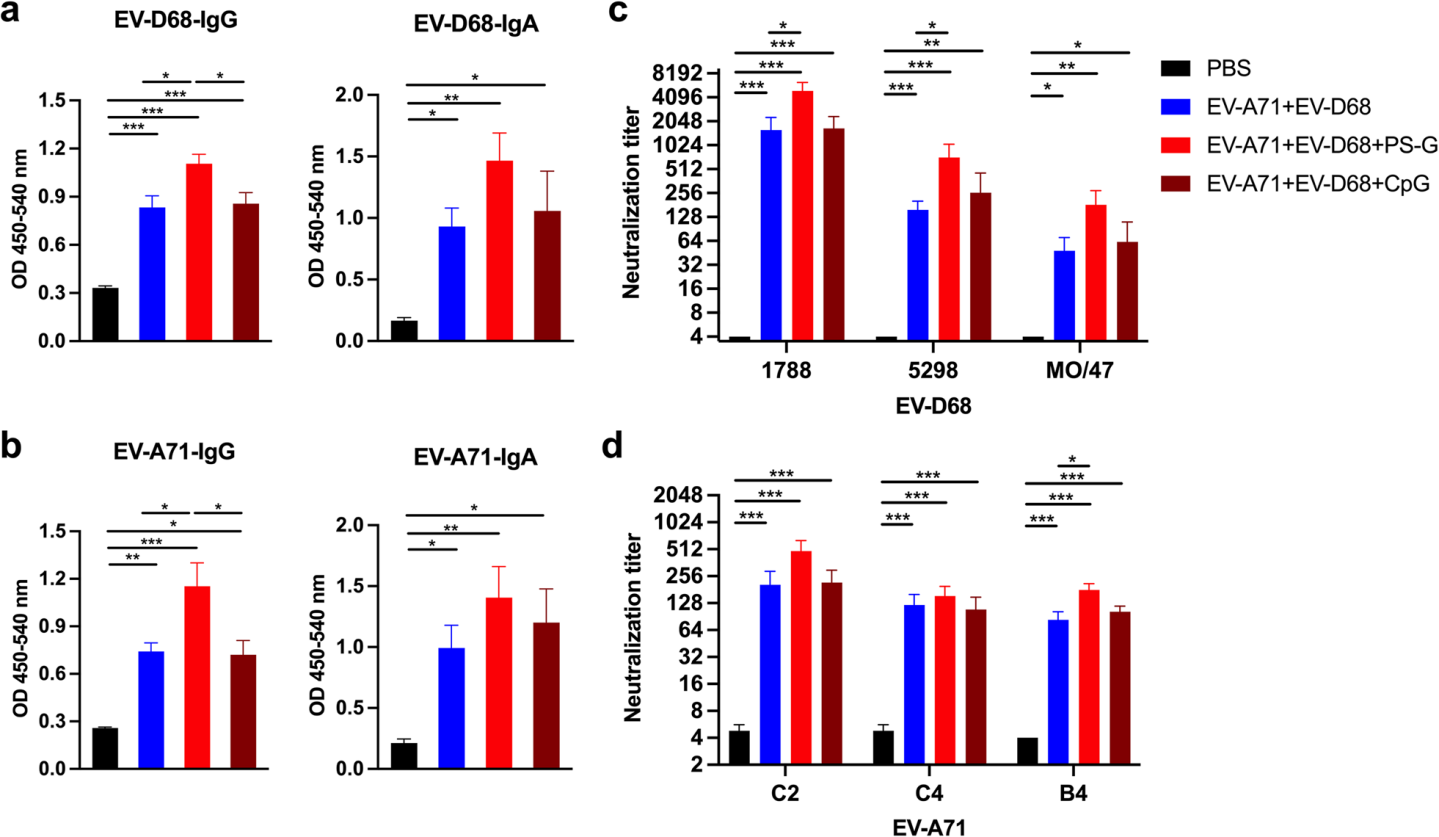
Effect of the EV-A71 + EV-D68 bivalent vaccine with or without PS-G or CpG as adjuvant on the levels of EV-D68- and EV-A71-specific IgG and IgA, and neutralization titre against infection by different enteroviruses in the sera of mice. The levels of **a** EV-D68-specific IgG and IgA and **b** EV-A71-specific IgG and IgA in the sera of mice after the third intranasal immunization were measured using ELISA. Sera were serially diluted (2^3^–2^12^), mixed with **c** EV-D68 and **d** EV-A71 virus, and used to infect RD cells. After 4 d, the highest dilution that resulted in the virus producing no cytopathic effect was considered to be the neutralization titre. All data are expressed as the mean ± SEM. *p < 0.05, **p < 0.01, ***p < 0.001

We observed that compared with the PBS group, mice vaccinated with the EV-A71 + EV-D68, EV-A71 + EV-D68 + PS-G, and EV-A71 + EV-D68 + CpG vaccines generated a considerable amount of EV-D68-specific (Fig. 6a) and EV-A71-specific (Fig. 6b) IgG and IgA in the sera after the third intranasal immunization. Compared with the EV-A71 + EV-D68 vaccine group alone, mice vaccinated with the EV-A71 + EV-D68 vaccine using PS-G as an adjuvant generated a significant amount of EV-D68-specific (*p* < 0.05) (Fig. 6a) and EV-A71-specific (*p* < 0.05) (Fig. 6b) IgG and IgA in the sera after the third intranasal immunization. Interestingly, we detected that the levels of the EV-A71- and EV-D68-specific IgG in the sera were higher in the group inoculated with the bivalent vaccine with PS-G as an adjuvant than that with CpG. The utilization of CpG as an adjuvant yielded results akin to those observed in mice immunized with the bivalent vaccine without an adjuvant.

To determine whether the inactivated EV-A71 + EV-D68 vaccine could elicit functional anti-EV-D68 and anti-EV-A71 antibodies, we analysed the neutralizing antibody titre in the sera after the third vaccination using a TCID_50_-reduction assay in RD cells. As previously mentioned, we serially diluted sera two-fold and cultured it with EV-D68 or EV-A71 in RD cells, and then determined the neutralizing titre as the highest dilution that neutralized 100-fold TCID_50_ of EV-D68 or EV-A71 virus, resulting in no cytopathic effects in RD cells. All mice vaccinated with the inactivated EV-A71 + EV-D68, EV-A71 + EV-D68 + PS-G, and EV-A71 + EV-D68 + CpG vaccines generated antibodies capable of neutralizing different EV-D68 (Fig. 6c) and EV-A71 sub-genotypes (Fig. 6d) in the sera after the third vaccination. Interestingly, the EV-A71 + EV-D68 + PS-G group showed a significantly higher cross-neutralization titre in the sera after the third vaccination compared with that in the EV-A71 + EV-D68 group (*p* < 0.05, EV-D68 5298;* p* < 0.05, EV-A71 B4). In addition, the EV-A71 + EV-D68 + PS-G group showed a higher cross-neutralization titre than that of the EV-A71 + EV-D68 + CpG group.

### *Cellular immune responses in EV-A71* + *EV-D68 bivalent vaccine immunization using PS-G as an adjuvant*

**Fig. 7:**
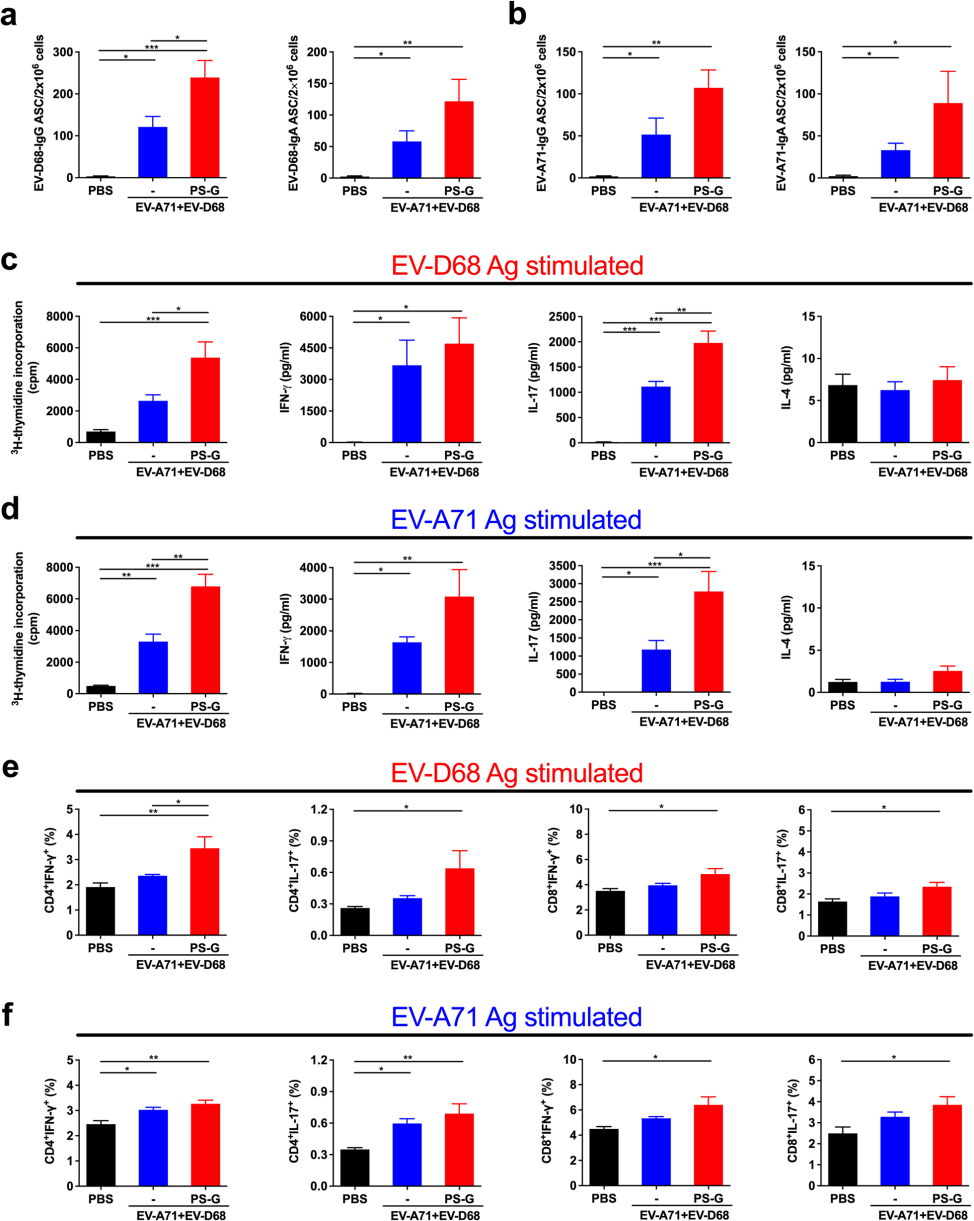
Effect of the EV-A71 + EV-D68 bivalent vaccine with or without PS-G as adjuvant on the number of antibody-secreting cells (ASCs), T-cell proliferation, and cytokine release in splenocytes. Mice were intranasally immunized with PBS, formalin-inactivated EV-A71 (2.5 μg/mouse) and EV-D68 (2.5 μg/mouse), and formalin-inactivated EV-A71 (2.5 μg/mouse) and EV-D68 (2.5 μg/mouse) combined with PS-G (10 μg/mouse) as adjuvant thrice at 3-week intervals. Spleen was isolated at 2 weeks after the third immunization, and then the numbers of **a** EV-D68-specific IgG and IgA ASCs and **b** EV-A71-specific IgG and IgA ASCs were measured using ELISPOT. **c**, **d** Splenocytes from immunized mice were harvested and cultured in medium containing 10 μg/mL heat-inactivated EV-D68 or EV-A71. After culture for 5 d, proliferation was measured as [^3^H]-thymidine incorporation in splenocytes. Culture supernatants of splenocytes were analysed for the levels of IFN-γ, IL-17, and IL-4 after 2 d in response to heat-inactivated EV-D68 or EV-A71. **e**, **f** Information on IFN-γ and IL-17 production by CD4^+^ and CD8^+^ T-cells in the spleen were obtained from all mice. Splenocytes were collected from mice immunized with PBS, formalin-inactivated EV-A71 + EV-D68, or PS-G-adjuvanted EV-A71 + EV-D68 and cultured in RPMI medium containing 10 μg/mL of heat-inactivated EV-A71 or EV-D68. Cells were cultured for 5 d, and the levels of IFN-γ and IL-17 produced by CD4^+^ and CD8^+^ T-cells were measured by flow cytometry. All data are expressed as the mean ± SEM. *p < 0.05, **p < 0.01, ***p < 0.001

To characterize the quality of memory responses induced by PS-G-adjuvanted EV-A71 + EV-D68 bivalent vaccines, we evaluated the number of antibody-secreting B-cells in the spleen. We found that compared with the PBS group, the number of EV-D68-specific IgG ASCs and that of EV-D68-specific IgA ASCs was significantly increased in mice vaccinated with EV-A71 + EV-D68 (*p* < 0.05) and EV-A71 + EV-D68 plus PS-G (*p* < 0.001) (Fig. 7a). Likewise, the number of EV-D68-specific IgG ASCs in mice vaccinated with EV-A71 + EV-D68 plus PS-G was significantly higher than that in the EV-A71 + EV-D68 group (*p* < 0.05) (Fig. 7a). We also noticed that compared with the PBS group, the numbers of EV-A71-specific IgG and IgA ASCs were significantly increased in mice vaccinated with EV-A71 + EV-D68 or the EV-A71 + EV-D68 + PS-G bivalent vaccine (*p* < 0.05) (Fig. 7b).

Regarding T-cell responses, we detected the enhanced proliferation and activation of T-cells in response to EV-D68 or EV-A71 antigen in mice immunized with the EV-A71 + EV-D68 or EV-A71 + EV-D68 + PS-G bivalent vaccine (Fig. 7c). We also measured the production of IFN-γ, IL-17, and IL-4 by splenocytes stimulated with EV-D68 or EV-A71 antigens using ELISA. We accordingly found that compared with the PBS group, EV-D68-stimulated splenocytes from mice vaccinated with EV-A71 + EV-D68 or EV-A71 + EV-D68 + PS-G bivalent vaccines produced considerably higher levels of IFN-γ and IL-17 (Fig. 7c). Compared with the EV-A71 + EV-D68 vaccine group, splenocytes from mice vaccinated with EV-A71 + EV-D68 + PS-G produced significantly higher levels of IL-17 (*p* < 0.01) (Fig. 7c). Similarly, we detected that compared with the PBS group, EV-A71-stimulated splenocytes from mice vaccinated with EV-A71 + EV-D68 or EV-A71 + EV-D68 + PS-G bivalent vaccines produced considerably higher levels of IFN-γ and IL-17 (Fig. 7d). Compared with the EV-A71 + EV-D68 vaccine group, splenocytes from mice vaccinated with EV-A71 + EV-D68 + PS-G produced significantly higher levels of IL-17 (*p* < 0.05) (Fig. 7d). However, we observed very low levels of IL-4 that did not differ between groups. In this study, PS-G enhanced adaptive Th1 and Th17 responses, but not Th2 responses.

To further confirm the immune activation mechanism of EV-A71 and EV-D68 mucosal bivalent vaccines, we examined the responses of CD4^+^ and CD8^+^ T-cells in immunized mice. We collected splenocytes from mice vaccinated with formalin-inactivated EV-A71 + EV-D68 with or without PS-G as adjuvant, and cultured them with heat-inactivated EV-D68 or EV-A71 in RPMI-1640 medium supplemented with 10% FBS. After incubating cells for 3 d, we determined the production of IFN-γ and IL-17 by CD4^+^ and CD8^+^ T-cells using flow cytometry (Fig. 7). We detected that significant levels of IFN-γ and IL-17 were secreted by CD4^+^ and CD8^+^ T-cells in heat-inactivated EV-D68 antigen-stimulated splenocytes from EV-A71 + EV-D68 + PS-G-vaccinated mice (*p* < 0.05) (Fig. 7e). We also found that significant levels of IFN-γ and IL-17 were released by CD4^+^ and CD8^+^ T-cells in heat-inactivated EV-A71 antigen-stimulated splenocytes from EV-A71 + EV-D68 + PS-G-vaccinated mice (*p* < 0.05) (Fig. 7f). These findings demonstrated that both CD4^+^ and CD8^+^ T-cells are involved in the anti-viral protection mediated by the EV-A71 + EV-D68 + PS-G vaccine. Moreover, they suggested that PS-G, as an adjuvant, is better at inducing cellular immune responses.

### In vivo protective assay of EV-A71 + EV-D68 bivalent vaccine immunization using PS-G as an adjuvant

**Fig. 8:**
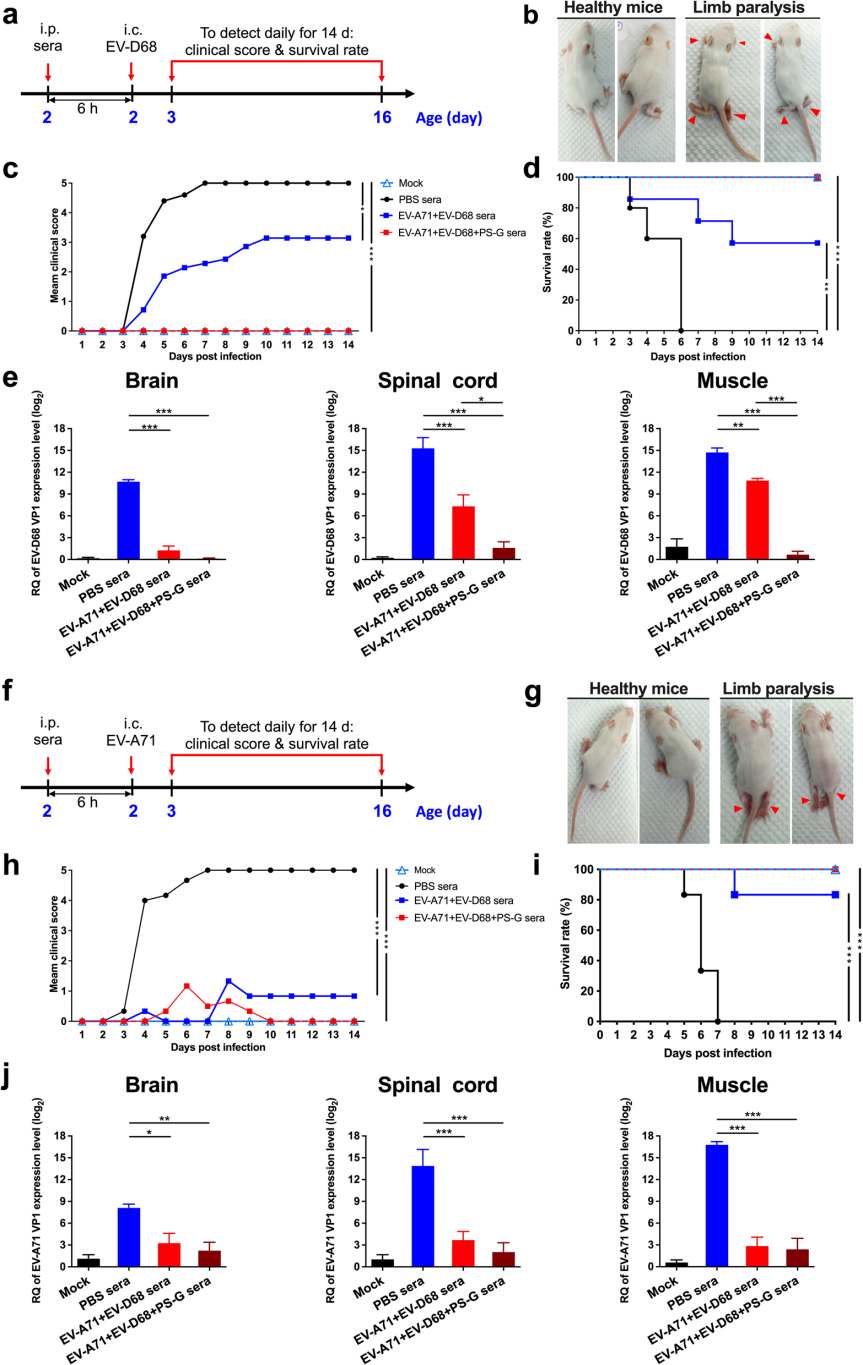
Protective efficacy of the inactivated EV-A71 + EV-D68 vaccine with or without PS-G as adjuvant in the EV-D68 or EV-A71 infection mouse model. The passive sera transfer and virus challenge schedules are shown. Briefly, 2-d-old ICR mice were i.p. injected with 20 μL anti-PBS, anti-EV-A71 + EV-D68, or anti-EV-A71 + EV-D68 + PS-G sera. After 6 h, the suckling mice were intracerebrally (i.c.) administered with RD medium, **a** EV-D68 (CDC_NO 2016-05298), or **f** EV-A71 (MP4). Mock mice were given medium only. **b**, **g** Representative images showing symptoms of healthy mice in the EV-A71 + EV-D68 or EV-A71 + EV-D68 + PS-G immunized group and mice with limb paralysis in the PBS-immunized group on day 9 post-infection with EV-D68 or EV-A71. Mice were monitored daily for **c**, **h** clinical score and **d**, **i** survival rates for 14 d following infection. Clinical scores were graded as described in “Methods”. **e**, **j** Brain, spinal cord, and muscle were collected after infection, and viral loads within the indicated tissues were determined using real-time quantitative PCR (qRT-PCR). The levels of expression were normalized to those of *GAPDH*. All data are expressed as the mean ± SEM. *p < 0.05, **p < 0.01, ***p < 0.001

We tested the in vivo protective efficacy of the EV-A71 + EV-D68 bivalent vaccine with or without PS-G as an adjuvant using a passive serum transfer assay. We i.p. injected 2-d-old ICR mice with anti-PBS, anti-EV-A71 + EV-D68, or anti-EV-A71 + EV-D68 + PS-G sera. After 6 h, we i.c. injected the suckling mice with a lethal dose of EV-D68 (5298) (Fig. 8a). We monitored the survival and clinical scores of mice daily for 14 d. Figure 8b shows representative images of protected mice in the EV-A71 + EV-D68- or EV-A71 + EV-D68 + PS-G-immunized group with healthy limbs and in the PBS-immunized group with limb paralysis on day 9 post-infection with EV-D68. We observed that the clinical score in the EV-A71 + EV-D68 group was remarkably lower than that in the PBS group, whereas it was zero in the EV-A71 + EV-D68 + PS-G group during the 2-week observation period (Fig. 8c). As shown in Fig. 8d, mice receiving anti-PBS sera started to show disease symptoms at 3 dpi and all of them eventually died within 6 dpi. In contrast, the survival rate of all of the anti-EV-D68 and EV-A71 sera-treated mice was 57.1% in the EV-A71 + EV-D68 group (*p* < 0.01) and 100% in the EV-A71 + EV-D68 + PS-G group (*p* < 0.001). To study the replication and distribution of EV-D68 in infected mice, we determined the viral loads in the brain, spinal cord, and muscle tissues of EV-D68 (5298)-infected mice using real-time PCR. We found that the levels of EV-D68 VP1-specific RNA in the brain, spinal cord, and muscles were considerably decreased in the EV-A71 + EV-D68 and EV-A71 + EV-D68 + PS-G groups (Fig. 8e). In addition, the levels of EV-D68 VP1-specific RNA in these tissues in the EV-A71 + EV-D68 + PS-G group were lower than those in the EV-A71 + EV-D68 group.

We i.p. injected newborn ICR mice with anti-PBS, anti-EV-A71 + EV-D68, or anti-EV-A71 + EV-D68 + PS-G sera, and then challenged them with a lethal dose of EV-A71 (MP4) (Fig. 8f). Figure 8g shows representative images of protected mice in the EV-A71 + EV-D68- or EV-A71 + EV-D68 + PS-G-immunized groups with healthy limbs and in the PBS-immunized group with limb paralysis on day 9 post-infection with EV-A71. Mice i.c. injected with RD medium alone (mock) did not show any indicators of disease, and all survived. We noticed that the clinical scores in the EV-A71 + EV-D68 and EV-A71 + EV-D68 + PS-G groups were considerably lower than those in the PBS group during the 2-week period (Fig. 8h). Mice receiving anti-PBS sera started to exhibit disease symptoms at 4 dpi, and all of them eventually died within 7 dpi. In contrast, the survival rate of antisera-treated mice was 83.3% in the EV-A71 + EV-D68 group (*p* < 0.001) and 100% in the EV-A71 + EV-D68 + PS-G group (*p* < 0.001) (Fig. 8i). Evaluation of the replication and distribution of EV-A71 in infected mice revealed that the levels of EV-A71 VP1-specific RNA in the brain, spinal cord, and muscles were considerably decreased in the EV-A71 + EV-D68 + PS-G group (Fig. 8j).

These results indicated that passive transfer of anti-EV-A71 + EV-D68 or anti-EV-A71 + EV-D68 + PS-G sera could provide effective protection against lethal EV-D68 or EV-A71 challenges in a mouse model.

## Discussion

In recent years, non-polio EVs, especially EV-A71 and EV-D68, have affected millions of people, emerging as the principal causes of EV-associated acute flaccid myelitis and paralysis, with sometimes lethal consequences. Three EV-A71 vaccines (inactivated whole virus C4 genotype) and two EV-A71 vaccines (inactivated whole virus B4 genotype) have been licensed for use in China and Taiwan, respectively [24, 25]. To date, there is no approved vaccine for EV-D68, and no anti-viral drugs have been approved to treat EV-A71 and EV-D68 infections. In the future, the number of people infected by EV-D68 may gradually increase. Therefore, it is very important to develop an EV-D68 vaccine, especially an EV-A71/EV-D68 bivalent vaccine, to ensure the effective prevention of AFM and AFP.

Mucosal surfaces are the first sites of entry for most pathogens. Accordingly, the induction of effective mucosal immunity is the first line of host defence against pathogens. Mucosal vaccination has the advantages of simple production, easy accessibility, large-scale vaccination, low cost, and being needle-free. Mucosal vaccination induces both mucosal and systemic immune responses. Vaccination via the nasal mucosa is easy and has been extensively investigated in animal models and clinical studies [30]. Several intranasal severe acute respiratory syndrome coronavirus 2 (SARS-CoV-2) vaccine candidates, including viral and protein subunit vaccines, are currently in phase III clinical trials [31]. The nasal cavity contains a large population of DCs, T-cells, and B-cells covered by an epithelial layer containing unique cells called microfold cells [32]. Immune responses in the respiratory tract are mediated by the nasal-associated lymphoid tissue [33]. Recently, our group demonstrated that inactivated whole virus-based EV-A71 mucosal vaccines with CpG or PS-G as adjuvants showed protective efficacy in animal models [23, 29]. These results led us to develop a bivalent mucosal vaccine comprising inactivated EV-A71 and EV-D68.

The development of bivalent or multivalent vaccines often requires attention to the phenomenon of immune interference, in which one immunogen in a combination vaccine is immunodominant over the other, resulting in an imbalance in the immune response and protection against target pathogens [34, 35]. In this study, we aimed to determine whether co-immunization with inactivated EV-A71 and EV-D68 via the nasal route can evoke a balanced immune response and protect against both viruses. Our data demonstrated that the bivalent vaccine was able to induce mucosal secretory IgA (sIgA) and serum IgG antibodies at levels similar to those induced by the corresponding monovalent vaccines, and the mucosal sIgA and serum IgG antibodies from the monovalent vaccine groups displaying no cross-reactivity with heterologous antigens (Fig. 2b–e). We also found that the bivalent vaccine elicited serum antibodies that neutralized both EV-A71 and EV-D68 at levels similar to those induced by the corresponding monovalent vaccines, with serum antibodies effectively cross-neutralizing their corresponding sub-genotypes (Fig. 2f, g). However, the same sera did not neutralize the respective heterologous viruses. These results indicated the lack of immune interference between the two immunogens. In addition, the antisera from the bivalent vaccine provided protection against lethal infection with either EV-D68 or EV-A71, whereas the two monovalent vaccines protected only against their respective homologous, but not heterologous, viral infections (Fig. 4). These data demonstrated that a bivalent vaccine candidate containing inactivated EV-A71 and EV-D68 antigens can confer balanced protective immunity against EV-A71 and EV-D68 infections, thus allowing the development of an inactivated whole-virus-based bivalent vaccine for both EV-A71 and EV-D68. The bivalent vaccine has increased protection relative to the monovalent vaccines in this neonatal mouse models. Several plausible explanations underpin this phenomenon: (i) Adjuvant effects: the presence of both EV-A71 and EV-D68 antigens in the bivalent vaccine may enhance the antigen presentation and activation of immune cells, leading to a more potent immune response compared with that of monovalent vaccines. (ii) Complementary immune mechanisms: EV-A71 and EV-D68 might elicit slightly different immune responses due to differences in their antigens or interactions with the immune system. The combination of both viruses within the bivalent vaccine could trigger a synergistic or complementary immune response. (iii) Synergistic Fc-mediated effector functions: the bivalent vaccine holds the potential to trigger synergistic Fc-mediated effector functions. Collectively, these factors allow for better protection against both individual viruses in the bivalent mucosal vaccine.

The NT titres for EV-D68 (1788) were much higher than those for EV-A71 (C2). The reason for this may be because EV-D68 (1788) potentially possess epitopes that are more readily accessible to neutralizing antibodies elicited in EV-D68 (1788)-immunized mice. In this study, the vaccine antigens employed were EV-A71 (TW/2272/98) and EV-D68 (CDC_NO 2010-01788). However, these two viruses were unable to cause death in neonatal mice at high titres (Additional file 1: Figure S2). For the viral challenging assays of the mouse model (Additional file 1: Figure S2), we used EV-A71 (TW/4643/MP4) and EV-D68 (CDC_NO 2016-05298), which are distinct from those used as vaccine antigens. Therefore, the protective efficacy of EV-D68 (1788)-immunized sera against EV-D68 (5298) infection in challenged mice was comparatively diminished. The in vivo environment is complex and involves interactions between viral and host factors. The complexity of immune responses and protection against viruses are influenced by multiple factors beyond NT titres alone [36]. The lack of a direct correlation between NT titres and protection levels may be attributed to various factors, including variances in viral strains, variations in the composition of anti-viral proteins within the sera, presence of non-neutralization antibodies, and potential contributions of Fc-mediated effector functions in bolstering protection.

Adjuvants have long been used in vaccines to promote immune responses, reduce vaccine dosage, and production costs. Despite the development of many vaccine adjuvants, their use in human vaccines is limited owing to their limited efficacy or side effects. Therefore, it is important to develop new vaccine adjuvants. Many studies have shown that natural polysaccharides derived from traditional Chinese medicine have good immune-promoting effects and can simultaneously improve humoral, cellular, and mucosal immunity. Mucoadhesive polysaccharide adjuvants have recently attracted increased attention in the field of vaccine development because of their advantages of protecting antigens from degradation, prolonging their residence time at target sites, controlling the release of loaded vaccines, low toxicity, and safety [37]. Dendritic cells play a critical role in determining the magnitude of the vaccination-induced immune response. As antigens alone do not usually induce a strong response, the addition of appropriate adjuvants is a common strategy for enhancing the immunogenicity of vaccine antigens [38]. Reishi has been reported to effectively modulate immune functions. Many studies have demonstrated that *Ganoderma lucidum* can stimulate T lymphocytes and macrophages [28], increase the activity of several kinases, inhibit neutrophil apoptosis, and enhance the phagocytic activity and migration ability of primary human neutrophils [39–41]. In previous studies, we have demonstrated that polysaccharides from the fruiting body of *G. lucidum* (PS-G) can induce the activation and maturation of human DCs via the NF-κB and p38 mitogen-activated protein kinase pathways [21], as well as IL-12 secretion, thus inducing Th1-related cytokine and chemokine production [22]. IL-12 plays an important role in linking the innate and adaptive immune systems. PS-G is also an active adjuvant that can stimulate the Th1 response and antibody production via the intraperitoneal route [21]. *Ganoderma lucidum* polysaccharide (GLP) was shown to exert strong adjuvant activity against Newcastle disease by promoting the activation and maturation of DCs [42]. In addition, GLP liposomes showed excellent adjuvant activity, potentiating the immunogenicity of porcine circovirus type-II [43]. Recently, we also demonstrated that PS-G-adjuvanted EV-A71 could effectively induce an EV-A71-specific immune response against infection by EV-A71 virions via the intranasal route [23].

In this bivalent enterovirus mucosal vaccine, we used CpG as an alternative adjuvant for comparison with PS-G. Our results showed that the EV-A71- and EV-D68-specific IgG and IgA titres in the serum, saliva, nasal wash, BALF, and feces, as well as the neutralization titres were substantially higher in the bivalent vaccine with PS-G as an adjuvant compared with those in the vaccine with CpG. The utilization of CpG as an adjuvant yielded results akin to those observed in the bivalent vaccine without an adjuvant. Therefore, our findings indicated that CpG was not a suitable choice for the enterovirus bivalent mucosal vaccine. Thus, we decided to focus our efforts specifically on PS-G as the preferred adjuvant. It is pertinent to note that while our prior research demonstrated the efficacy of CpG as an adjuvant in monovalent EV-A71 mucosal vaccines [29], CpG was not effective in the bivalent enterovirus mucosal vaccine in this study, probably due to the different antigenic characteristics and complexity of vaccines. Another reason may be that the concentration of CpG was not sufficient to activate the immune response, or the concentration was too high, leading to immune tolerance and weakening the immune response.

In this study, we confirmed that intranasal vaccination induced EV-A71- and EV-D68-specific sIgA not only in the respiratory tract but also at other mucosal sites, such as the intestinal tract. Mucous membranes are widely distributed in the human body, and different parts of the mucosal surface can connect with each other through lymphocyte homing, guiding the transmission of the immune response at the mucosal induction site to distant mucosal effect sites [44]. Formalin-inactivated EV-A71/EV-D68 with PS-G as a mucosal adjuvant was demonstrated to be an excellent vaccine because it not only induced high titres of neutralizing antibodies to prevent lethal viral challenge but also induced the production of sIgA antibodies, demonstrating that immunization through the mucosal route produces both a mucosal and systemic immune response. More importantly, the formalin-inactivated mucosal vaccine based on the EV-A71 C2 and EV-D68 B3 genotypes not only induced high levels of antibodies against the same EV-A71 C2 and EV-D68 B3 genotypes but also against the B4 and C4 genogroups of EV-A71 and B1 of EV-D68 (MO/47), indicating the broad cross-protection of the vaccine against EV-A71 and EV-D68 strains of different genotypes (Figs. 2f, g and 6c, d).

In the assessment of immunogenicity, antibody-producing cells serve as critical indicators of immunological memory on mucosal surfaces. Our results demonstrated an increased number of EV-A71- and EV-D68-specific IgA and IgG antibody-producing cells in the splenocytes of mice immunized with the PS-G-adjuvanted EV-A71 + EV-D68 vaccine. These data correlated with the EV-A71- and EV-E68-specific IgA and IgG antibody titres in the sera and mucosal tissues, suggesting that PS-G promotes B-cell proliferation and enhances humoral immunity. In addition to T lymphocyte immunity, when the EV-A71/EV-D68 vaccine was formulated with PS-G as an adjuvant, T-cell proliferative responses, as well as IFN-γ and IL-17 secretion in splenocytes were all significantly increased. These results demonstrated that EV-A71/EV-D68 mucosal immunity greatly stimulated the Th1 and Th17 T-cell responses and triggered a humoral Th1 serum IgG2c response (Additional file 1: Figure S1). Recent studies have indicated that Th17 cells, and their key cytokine IL-17, play pivotal roles in the induction of innate and adaptive host responses and contributing to host defence against pathogens at mucosal surfaces [45]. The benefits of the IL-17 response in vaccinated mice infected with EV-A71 or EV-D68 include the reinforcement of mucosal immunity, recruitment of immune cells, anti-viral activity, regulation of inflammatory responses, and potential tissue protection [46]. These combined effects can contribute to a stronger and more effective immune response against enterovirus infections. Using flow cytometry, we further found that both CD4^+^ and CD8^+^ T-cells were able to produce IFN-γ and IL-17 in splenocytes. Our data showed that the number of CD4^+^ and CD8^+^ T-cells was increased in the EV-A71 + EV-D68 + PS-G group. Activation of antigen-specific CD4^+^ and CD8^+^ T-cells is a major indicator of cellular immune response. Both CD4^+^ and CD8^+^ T-cells were reported to protect mice from EV-A71 infection by reducing the viral load in tissues [47]. CD8^+^ T-cells possess antigen-specific cytotoxic activity and can alter their own population by mounting a cytotoxic response that lyses the virus-infected cells. Evaluation of T-cell immunity provides a more comprehensive understanding of the immune response induced by the mucosal vaccine. A balanced immune response, involving both antibody production and T-cell activation, can contribute to robust and enduring protection against the virus. While neutralizing antibodies are critical, T-cells play a pivotal role in eliminating infected cells and facilitating long-term memory against the virus. Measuring both humoral and cellular immune responses provides a holistic perspective of the impact of the mucosal vaccine on the immune system.

The protective efficacy of the inactivated EV-A71/EV-D68 bivalent vaccine with or without PS-G as an adjuvant was tested using a passive serum immunization assay. The clinical score, survival rate, and viral RNA expression results demonstrated that the EV-A71 + EV-D68 + PS-G vaccine had a good protective effect against EV-A71 and EV-D68 viral infections in vivo (Fig. 8). In this protection model as only sera are transferred to the neonates, how PS-G elicits a greater immune response. Possible reasons include mainly the production of non-neutralizing antibodies. While neutralizing antibodies are traditionally considered critical for viral clearance, non-neutralizing antibodies can also play a significant role. These antibodies may still interact with viral proteins, affecting viral entry, replication, and clearance through various mechanisms. In addition, Fc-mediated effector functions play a role in infection protection by broadly neutralizing antibodies and may be also important for protection by non-neutralizing antibodies. This comprehensive spectrum of Fc-mediated effector functions encompasses mechanisms such as antibody-dependent cellular cytotoxicity, antibody-mediated complement activation, and antibody-dependent phagocytosis. Moreover, the presence of anti-viral proteins in the serum can interfere with viral replication and dissemination [48, 49]. Collectively, these multifaceted mechanisms synergize to confer superior protection against both individual viral infections. Taken together, these findings demonstrated that PS-G, as a potential adjuvant for the EV-A71/EV-D68 bivalent nasal vaccine, could induce immune cell responses and protect against lethal enterovirus challenge.

Vaccines represent vital tools for establishing long-term immune memory, which is essential in the prevention and control of infectious diseases. We demonstrated that monovalent EV-A71 or EV-D68 mucosal vaccines can induce EV-A71- or EV-D68-specific IgA and IgG responses in the serum, feces, and saliva, as well as the development of neutralizing titres in the serum, which persist for at least 16 or 19 weeks after the third dosage. Moreover, the administration of the fourth intranasal immunization with either EV-A71 or EV-D68 re-stimulated and significantly increased the levels of EV-A71- or EV-D68-specific IgA and IgG, showing that EV-A71- or EV-D68-specific IgA and IgG could persist in the mucosa and serum for a long time, and suggesting memory effects in the mucosal tissues. In future investigations, we intend to delve into the long-term immune memory conferred by bivalent mucosal vaccines and explore the resident memory B-cells and T-cells in mucosal tissues to confirm the advantages of mucosal vaccines.

## Conclusions

Our study not only demonstrated that EV-A71 and EV-D68 are promising candidate bivalent vaccines for enterovirus infection but also showed that PS-G is a novel adjuvant for the EV-A71/EV-D68 bivalent mucosal vaccine. Our results support the development and use of an intranasal EV-A71/EV-D68 bivalent vaccine for inducing systemic and mucosal immune responses in the clinic in the future.

### Supplementary Information

**Additional file 1: Figure S1.** EV-D68- and EV-A71-specific IgG1 and IgG2c responses in sera of mice. **Figure S2.** Comparative assessment of the virulence of diverse enterovirus strains, infection routes, and inoculation titres in ICR mice.

## Data Availability

All data generated or analysed during this study are included in this published article. The data analysed in the current study are available upon reasonable request.

## Abbreviations

EV-A71: Enterovirus A71
EV-D68: Enterovirus D68
HFMD: Hand-foot-and-mouth disease
AFM: Acute flaccid myelitis
AFP: Acute flaccid paralysis
PS-G: *Ganoderma lucidum* Polysaccharide
DC: Dendritic cell
RD: Rhabdomyosarcoma
