# Supplementary material for: A novel mucosal bivalent vaccine of EV-A71/EV-D68 adjuvanted with polysaccharides from Ganoderma lucidum protects mice against EV-A71 and EV-D68 lethal challenge
Source: J Biomed Sci. 2023 Dec 18;30:96. doi: 10.1186/s12929-023-00987-3 (PMC10729491; doi:10.1186/s12929-023-00987-3)
Supplement: Supplementary file 1 — Additional file 1: Figure S1. EV-D68- and EV-A71-specific IgG1 and IgG2c responses in sera of mice. Figure S2. Comparative assessment of the virulence of diverse enterovirus strains, infection routes, and inoculation titres in ICR mice. [file 12929_2023_987_MOESM1_ESM.docx]

**Additional information**

**
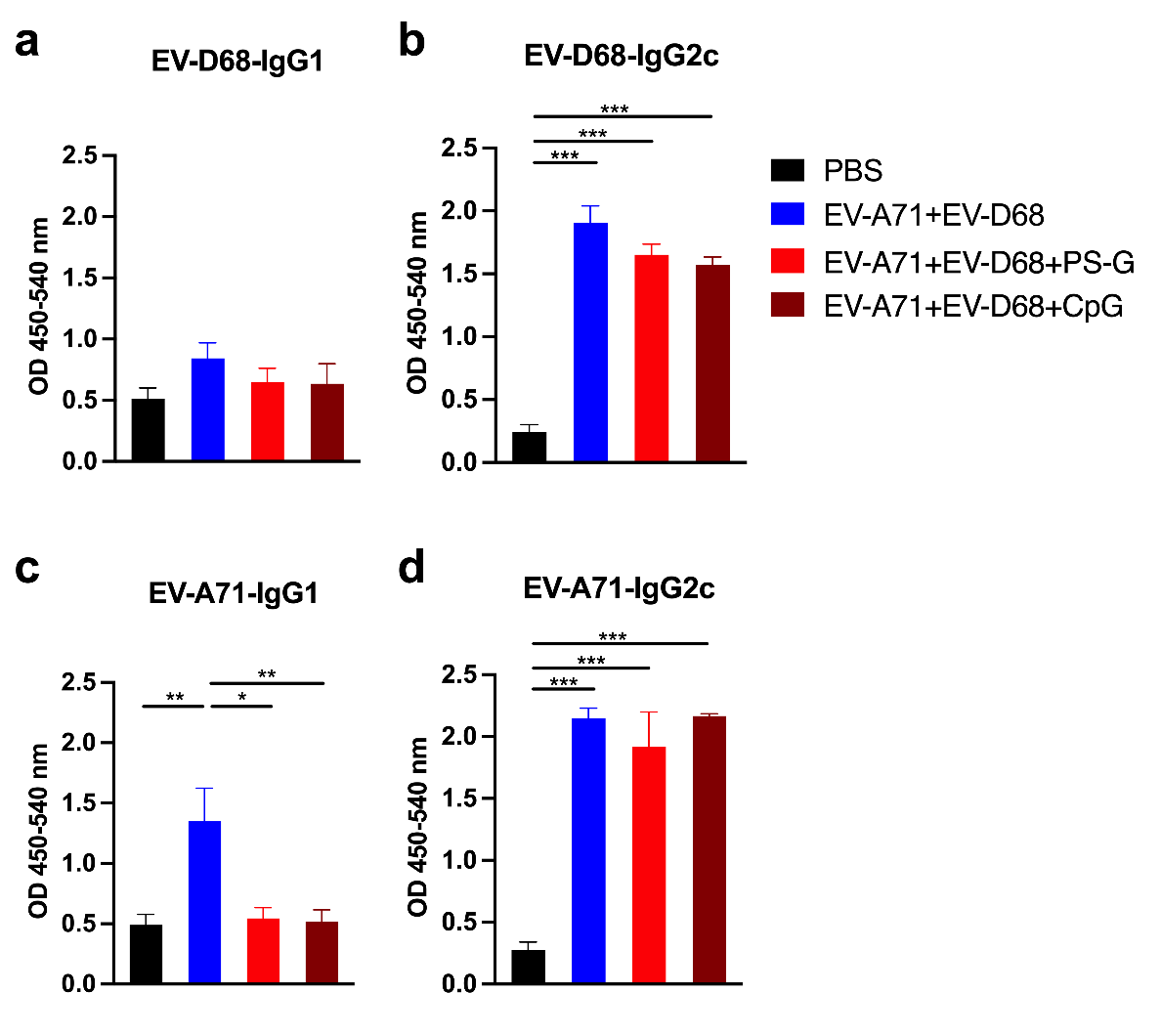
**

**Figure S1. EV-D68- and EV-A71-specific IgG1 and IgG2c responses in sera of mice.** Mice were intranasally immunized with PBS, formalin-inactivated EV-A71 (2.5 μg/mouse) and EV-D68 (2.5 μg/mouse), and formalin-inactivated EV-A71 (2.5 μg/mouse) and EV-D68 (2.5 μg/mouse) combined with PS-G (10 μg/mouse) or CpG (20 μg/mouse) as adjuvant thrice at 3-week intervals. The levels of EV-D68-specific IgG1 **(a)** and IgG2c **(b)**, and EV-A71-specific IgG1 **(c)** and IgG2c **(d)** in the sera of mice after the third immunization were measured using ELISA. All data are expressed as the mean ± SEM. **p* < 0.05, ***p* < 0.01, ****p* < 0.001.

**Results**

**EV-D68- and EV-A71-specific IgG1 and IgG2c responses to intranasal EV-A71+EV-D68 bivalent vaccine immunization using PS-G as an adjuvant**

To investigate the IgG subclass patterns of induced systemic humoral immune response, we analysed the serum levels of EV-D68- and EV-A71-specific IgG1 (representing the Th2 response) and IgG2c (representing the Th1 response) in immunized mice. Mice immunized thrice with the EV-A71+EV-D68 bivalent vaccine showed higher levels of EV-A71-IgG1 compared with those in other groups (Fig. S1 c). In mice vaccinated with EV-A71+EV-D68 with or without PS-G or CpG as an adjuvant, the production of EV-D68- and EV-A71-specific IgG2c was significantly higher (p < 0.001) than that in mice immunized with PBS (Fig. S1 b and d). Overall, our results suggested that the EV-A71+EV-D68 bivalent mucosal vaccine with or without PS-G or CpG as an adjuvant could effectively trigger a humoral Th1 serum IgG2c response.


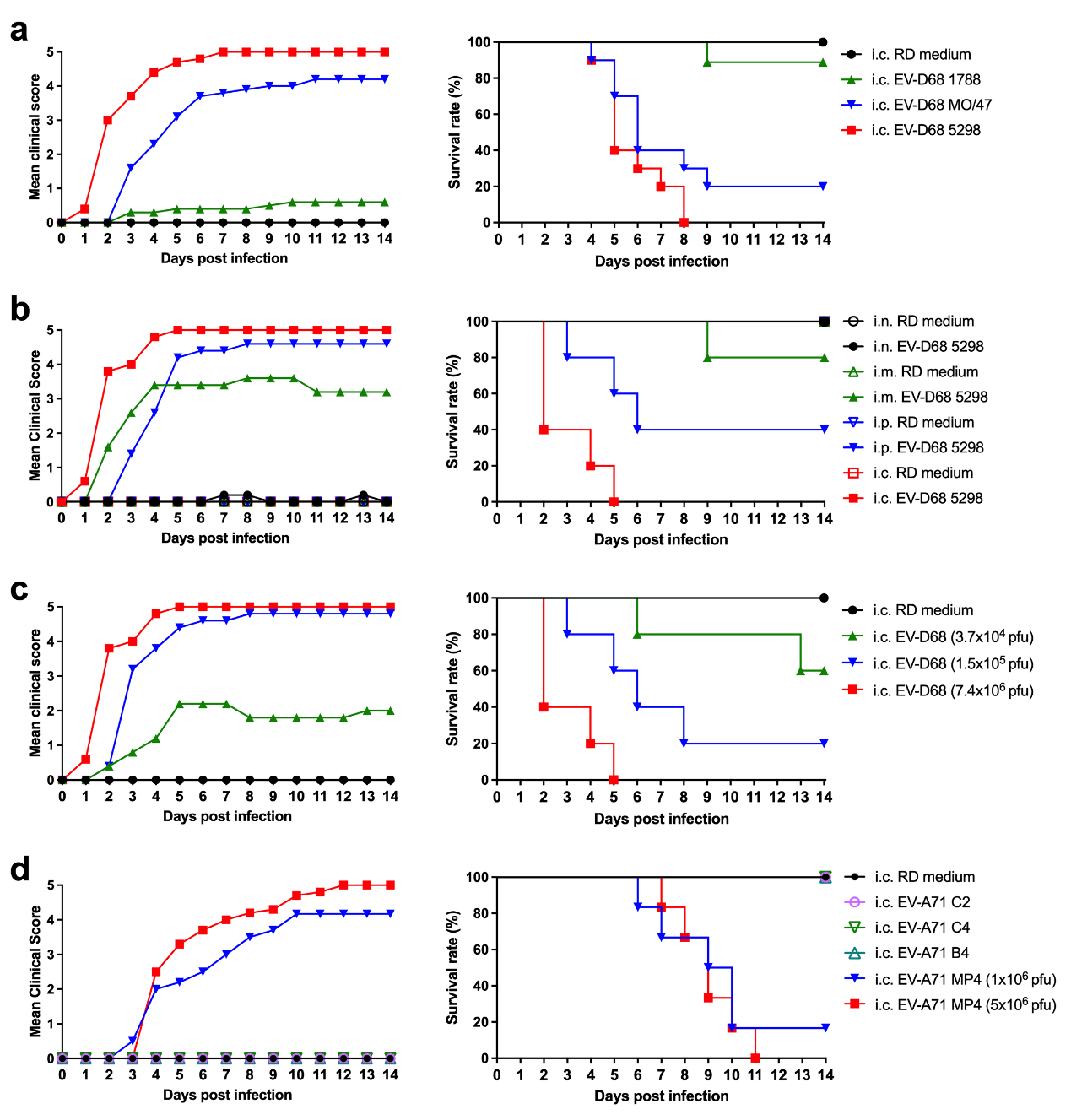


**Figure S2. Comparative assessment of the virulence of diverse enterovirus strains, infection routes, and inoculation titers in ICR mice.** Briefly, 2-d-old ICR mice were inoculated with (a, d) various EV-D68 and EV-A71 strains, (b) via intracranial (i.c.), intraperitoneal (i.p.), intramuscular (i.m.), and intranasal (i.n.) routes, and (c, d) varying inoculation titers. Control mice received RD medium only. The survival rates and clinical scores were then monitored and recorded daily after infection.

**Results**

**Assessment of the virulence of different enterovirus strains, infection routes, and inoculation titers in ICR mice**

Notably, EV-A71 and EV-D68 infections caused no apparent clinical symptoms in adult mice. Consequently, we used neonatal mice for our lethal challenge experiments. To substantiate the protective efficacy of the enterovirus mucosal vaccines, we established an Institute of Cancer Research (ICR) suckling mouse model of enterovirus infection with different EV-D68 and EV-A71 strains, infection routes, and inoculation titers in 2-d-old ICR mice. We observed a notable disparity in susceptibility among mice exposed to different infection routes, with intracranial inoculation resulting in the higher susceptibility compared with that exhibited by intraperitoneal, intramuscular, and intranasal routes. ICR neonatal mice were susceptible to infection with the EV-D68 5298 clinical strain and EV-A71/MP4 mouse-adapted strain via intracerebral injection. Notably, infected mice exhibited progressive limb paralysis prior to death. The mortality rate of mice was viral dose-dependent. Therefore, we used the EV-D68 5298 and EV-A71/MP4 strains via intracerebral injection as the preferred approach for our challenge model, thereby providing additional substantiation of the efficacy of the bivalent mucosal vaccine.
